# Crystal structures of five 6-mercaptopurine derivatives

**DOI:** 10.1107/S2056989016001833

**Published:** 2016-02-10

**Authors:** Lígia R. Gomes, John Nicolson Low, Diogo Magalhães e Silva, Fernando Cagide, Fernanda Borges

**Affiliations:** a FP–ENAS–Faculdade de Ciências de Saúde, Escola Superior de Saúde da UFP, Universidade Fernando Pessoa, Rua Carlos da Maia, 296, P-4200-150 Porto, Portugal; b REQUIMTE, Departamento de Química e Bioquímica, Faculdade de Ciências da Universidade do Porto, Rua do Campo Alegre, 687, P-4169-007, Porto, Portugal; c Department of Chemistry, University of Aberdeen, Meston Walk, Old Aberdeen AB24 3UE, Scotland; d CIQ/Departamento de Quιmica e Bioquιmica, Faculdade de Ciências, Universidade do Porto, 4169-007 Porto, Portugal

**Keywords:** crystal structure, mercaptopurines, supra­molecular structure

## Abstract

The crystal structures of five 6-mercaptopurine derivatives, *viz.* 2-[(9-acetyl-9*H*-purin-6-yl)sulfan­yl]-1-(3-meth­oxy­phen­yl)ethan-1-one (**1**), C_16_H_14_N_4_O_3_S, 2-[(9-acetyl-9*H*-purin-6-yl)sulfan­yl]-1-(4-meth­oxy­phen­yl)ethan-1-one (**2**), C_16_H_14_N_4_O_3_S, 2-[(9-acetyl-9*H*-purin-6-yl)sulfan­yl]-1-(4-chloro­phen­yl)ethan-1-one (**3**), C_15_H_11_ClN_4_O_2_S, 2-[(9-acetyl-9*H*-purin-6-yl)sulfan­yl]-1-(4-bromo­phen­yl)ethan-1-one (**4**), C_15_H_11_BrN_4_O_2_S, and 1-(3-meth­oxy­phen­yl)-2-[(9*H*-purin-6-yl)sulfan­yl]ethan-1-one (**5**), C_14_H_12_N_4_O_2_S. Compounds (**2**), (**3**) and (**4**) are isomorphous and accordingly their mol­ecular and supra­molecular structures are similar. An analysis of the dihedral angles between the purine and exocyclic phenyl rings show that the mol­ecules of (**1**) and (**5**) are essentially planar but that in the case of the three isomorphous compounds (**2**), (**3**) and (**4**), these rings are twisted by a dihedral angle of approximately 38°. With the exception of (**1**) all mol­ecules are linked by weak C—H⋯O hydrogen bonds in their crystals. There is π–π stacking in all compounds. A Cambridge Structural Database search revealed the existence of 11 deposited compounds containing the 1-phenyl-2-sulfanyl­ethanone scaffold; of these, only eight have a cyclic ring as substituent, the majority of these being heterocycles.

## Abstract 2

Three of five 6-mercaptopurine derivatives are isomorphous and accordingly their mol­ecular and supra­molecular structures are similar. In the remaining two derivatives, the purine and exocyclic phenyl rings are essentially planar, but that in the case of the three isomorphous compounds, these rings are twisted.

## Chemical context   

Purines, purine nucleosides and their analogs, are nitro­gen-containing heterocycles ubiquitous in nature and present in biological systems like man, plants and marine organisms (Legraverend, 2008 ▸). These types of heterocycles take part of the core structure of guanine and adenine in nucleic acids (DNA and RNA) being involved in diverse *in vivo* catabolic and anabolic metabolic pathways.

6-Mercaptopurine is a water insoluble purine analogue, which attracted attention due to its anti­tumor and immunosuppressive properties. The drug is used, among others, in the treatment of rheumathologic disorders, cancer and prevention of rejection of organ transplantation. The main problem associated with the pharmacological treatment with 6-mercaptopurine is the low bioavailability of the oral absorption and the short half-life in plasma. Strategies that have been adopted to circumvent those problems include the administration of 6-mercaptopurine analogues that act as prodrugs or by the chemical protection of the thiol group.

Chemically, the 6-mercaptopurine scaffold can also be modulated by an appropriate selection of the substituents that can be located at C-2, N-1, C-6, N-3, C-8, N-7 and N-9 positions, generating a variety of derivatives with potential biological applications (Legraverend & Grierson, 2006 ▸; Tunçbilek, *et al.*, 2009 ▸).

Within this framework, the goal of this project has been focused on the functionalization of 6-mercapto purine at positions 6 and 9. Here we describe the syntheses and characterization of five 6-mercaptopurine derivatives: 2-[(9-acetyl-9*H*-purin-6-yl)sulfan­yl]-1-(3-meth­oxy­phen­yl)ethan-1-one (**1**), 2-[(9-acetyl-9*H*-purin-6-yl)sulfan­yl]-1-(4-meth­oxy­phen­yl)ethan-1-one (**2**), 2-[(9-acetyl-9*H*-purin-6-yl)sulfan­yl]-1-(4-chloro­phen­yl)ethan-1-one (**3**), 2-[(9-acetyl-9*H*-purin-6-yl)sulf­an­yl]-1-(4-bromo­phen­yl)ethan-1-one (**4**) and 1-(3-meth­oxy­phen­yl)-2-[(9*H*-purin-6-yl)sulfan­yl]ethan-1-one (**5**).
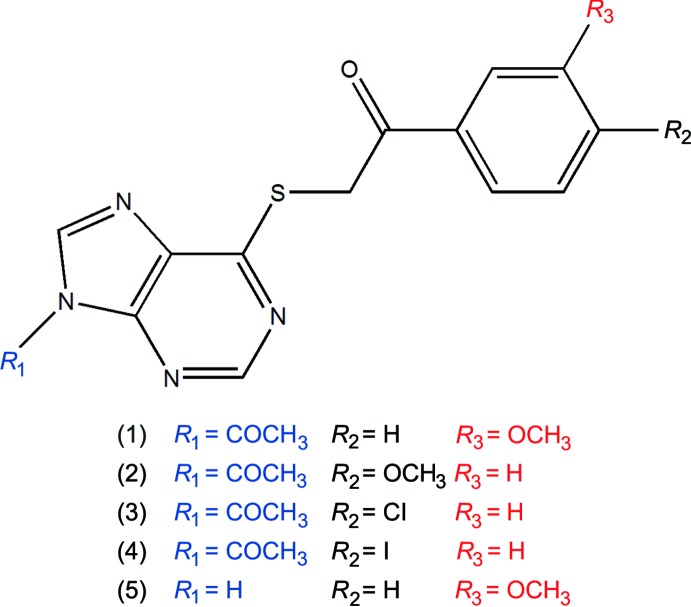


## Structural commentary   

**Figure 1:**
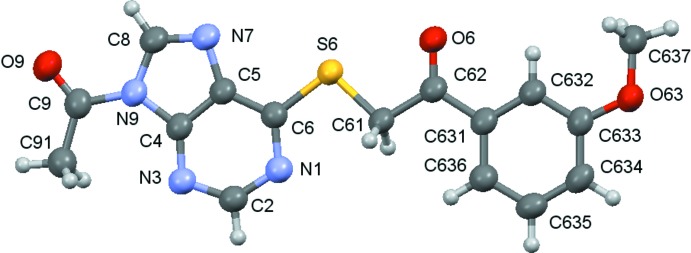
A view of the asymmetric unit of (**1**), with displacement ellipsoids are drawn at the 70% probability level.

Compounds (**1**)–(**5**) are shown in the scheme and their ellipsoid plots are shown in Figs. 1 ▸–5 ▸ ▸ ▸ ▸. Compounds (**1**) and (**5**) have similar *a* and *c* axes and (**2**), (**3**) and (**4**) are isostructural and isomorphous.

**Figure 6:**
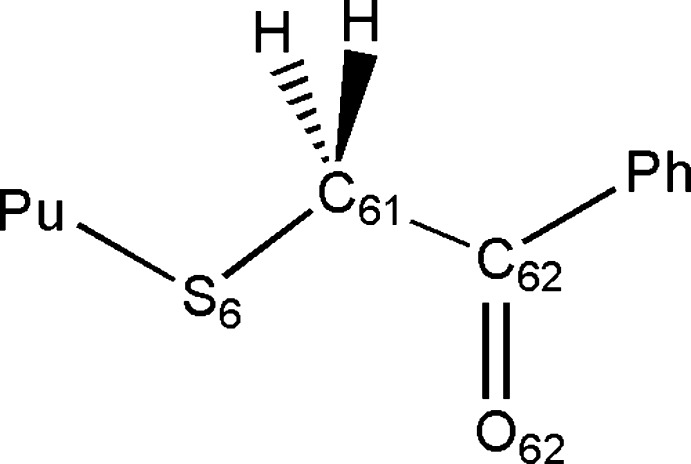
Diagram of the S–CH_2_–C(=O)– linkage.

These compounds can be envisaged as two building blocks, a substituted phenyl­ethanone grouping and a substituted 6-mercaptopurine moiety, bonded together by the mercapto ethanone residue. Since both purine and phenyl rings are essentially planar, the structural conformations of those compounds are conditioned by the –SCH_2_CO spacer (Fig. 6 ▸) which permits rotations around the following bonds: Pu—S6, S6—C61, C61—C62 and C62—Ph bonds. The *sp* ^3^ character of the central carbon atom may also direct the relative positions of the aceto­phenone residue out of the main plane constituted by the mercaptopurine, which is not the case of the present compounds. Selected geometric parameters for compounds (**1**)–(**5**) are given in Tables 1 ▸–5 ▸ ▸ ▸ ▸, respectively.

The Pu—S6 bond tends to be coplanar with the purine residue. In fact, the 6-mercaptopurine itself may appear in the thione form, *e.g.* 3,7-di­hydro­purine-6-thione, as a consequence of the high degree of electron delocalization within the 6-mercaptopurine environment. The tendency for the Pu—S6 bond to assume partial double-bond character is also seen in the present compounds, for which the corresponding Pu—S6 bond lengths lie between 1.741 (3) Å for (**2**) and 1.755 (3) Å for (**4**). In contrast, the S6—C61 bond lengths are longer, with values lying between 1.8017 (18) Å in (**1**) and 1.812 (3) Å in (**4**). This bond can also be bent with respect to the main mercaptopurine plane. The degree of bending may be evaluated by the distance of the C62 carbon atom from the mean plane consisting of the mercapto­pyrimidine atoms. Those values [0.307 (3), 0.272 (4), 0.333 (2), 0.332 (4) and 0.164 (2) for (**1**)–(**5**), respectively] show that the degree of bending is higher in (**1**)–(**4**) than in (**5**). As regards the ethanone group, the C61—C62 bond lengths lie in the range 1.510 (4) Å (**2**) to 1.528 (4) Å, (**4**) and are normal for a C*sp* ^3^—C*sp* ^3^ bond while the C62—Ph bond lengths are shorter and lie in the range 1.474 (3) Å (**2**) to 1.496 (4) (**4**), suggesting that the electron density is delocalized from the phenyl ring.

The dihedral angles between the mean planes of the of the purine and phenyl ring, θ_1_, those between the mean plane of the purine ring and the plane defined by the S6—C61—C62—O6 atoms, θ_2_, and those between the mean planes of the phenyl ring and the plane defined by the S6—C61—C62—O6 atoms, θ_3_ are given in Table 6 ▸. These values show that the mol­ecules of (**1**) and (**5**) are essentially planar. However, in the case of the three isomorphous compounds (**2**), (**3**) and (**4**), the purine and exocyclic phenyl rings are both twisted in the opposite direction from the plane of the bridging unit, resulting in a dihedral angle of approximately 38°. This is due to the rotations and bending around the bonds connecting the bridging unit to the purine and exocyclic phenyl rings as discussed above. The dihedral angles θ_2_ are higher than θ_3_; the former are mainly due to the rotations around the S6—C61 bond while the latter are mainly the result of the bending of the C62—Ph bond.

## Supra­molecular features   

**Figure 7:**
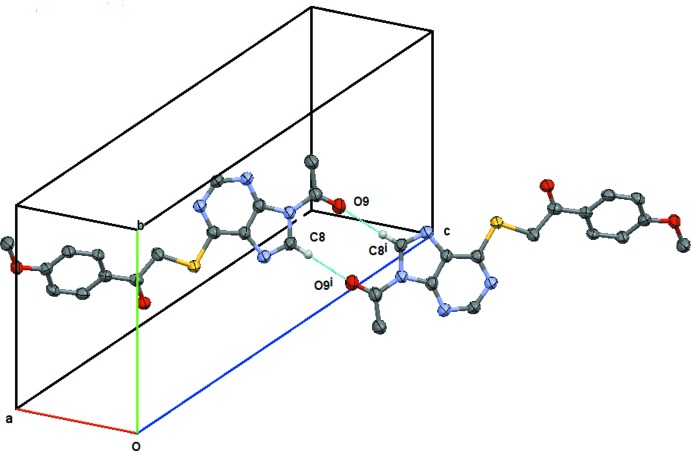
Compound (**2**): view of the C8—H8⋯O9 centrosymetric 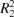
(16) ring structure centred on (−½, ½, −½). Symmetry code: (i) −*x* − 1, −*y* + 1, −*z* + 1. H atoms not involved in the hydrogen bonding are omitted.

**Figure 8:**
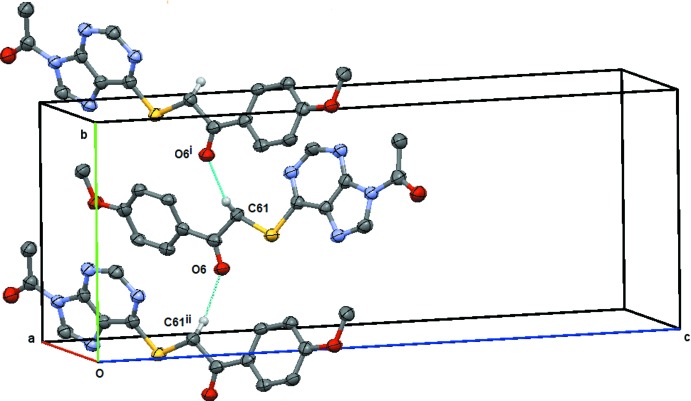
Compound (**2**): the simple *C*4 chain formed by the C61—H61*A*⋯O6 weak hydrogen bond. This chain extends along the *b* axis and is generated by the twofold screw axis at (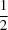, *y*, 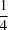). Symmetry codes: (i) −*x* + 1, *y* + 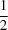, −*z* + 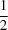; (ii) −*x* + 1, *y* − 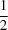, −*z* + 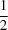. H atoms not involved in the hydrogen bonding are omitted.

**Figure 9:**
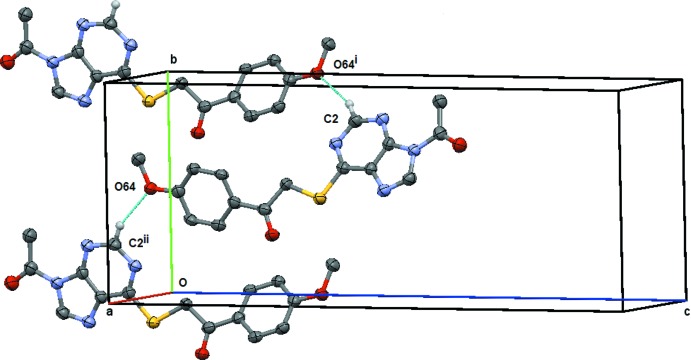
Compound (**2**): the simple *C*12 chain formed by the C2—H2⋯O64 weak hydrogen bond. This chain extends along the *b* axis and is generated by the twofold screw-axis at (1, *y*, 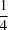). Symmetry codes: (i) −*x* + 2, *y* + 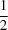, −*z* + 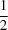; (ii) −*x* + 2, *y* − 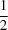, −*z* + 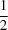. H atoms not involved in the hydrogen bonding are omitted.

**Figure 10:**
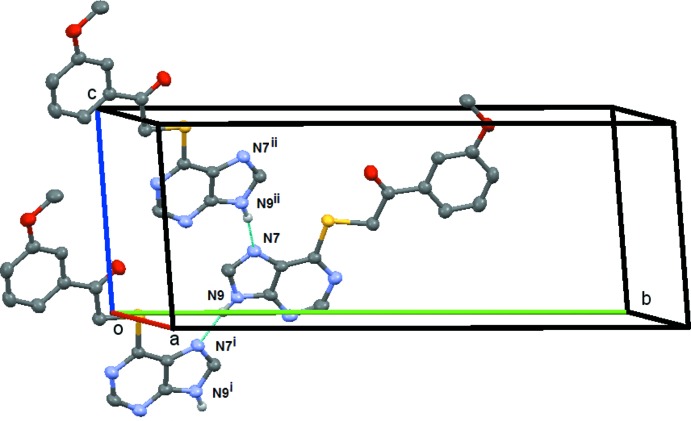
Compound (**5**): the simple *C*4 chain formed by the N9—H9⋯O64 weak hydrogen bond. This chain extends along the *b* axis and is generated by the *n*-glide plane at (0, 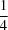, 0). Symmetry codes: (i) *x* − 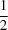, −*y* + 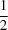, −*z* − 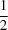; (ii) *x* − 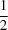, −*y* + 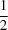, −*z* + 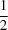. H atoms not involved in the hydrogen bonding are omitted.

There are no weak C—H⋯O or C—H⋯N contacts in (**1**). Hydrogen bonds for (**2**)–(**5**) are listed in Tables 7 ▸–10 ▸ ▸ ▸, respectively. Since (**2**), (**3**) and (**4**) are isomorphous, their supra­molecular structures follow similar patterns. Accordingly, hydrogen-bonding diagrams are given for (**2**) only. Atom C8 acts as a donor to O9 (−*x* − 1, −*y* + 1, −*z* + 1), *via* H8 forming an 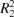
(10) centrosymmetric dimer across the inversion centre at (−1/2, 1/2, 1/2), Fig. 7 ▸. Atom C61 makes a hydrogen bond with O6 (−*x* + 1, *y* + 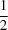, −*z* + 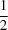), *via* H61*A*, forming a *C*4 chain, which runs parallel to the *b* axis, Fig. 8 ▸, generated by the twofold screw axis at (1/2, *y*, 1/4). In (**2**), there is a short contact between C6 and the 4-meth­oxy atom O64 (−*x* + 2, *y* + 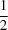, −*z* + 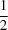), forming a *C*12 chain, Fig. 9 ▸, which runs parallel to the *b* axis and is generated by the twofold screw axis at (1, *y*, 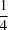). In (**5**), the N9—H9⋯N9 (*x* − 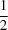, −*y* + 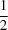, *z* − 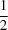) hydrogen bond, Fig. 10 ▸, links the mol­ecules into a *C*4 chain which runs parallel to [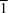
01] and which is generated by the *n*-glide plane at (0, 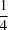, 0).

Since those compounds have three rings, the imidazole ring (with centroid *Cg*1), the pyrimidine ring (with centroid *Cg*2) and the benzyl ring (with centroid *Cg*3), it would be expected that π–π contacts were part of the supra­molecular structure. Table 11 ▸ lists the possible π–π contacts for (**1**)–(**5**). As may be seen in the Table, the pyrimidine ring establishes π–π contacts with the benzyl ring for all compounds. In (**1**), two mol­ecules centrosymmetrically related across the inversion centre at (0, ½, ½) are involved in π–π stacking in which the purine ring stacks above the exocyclic phenyl ring. In (**2**), (**3**) and (**4**), the π–π stacking is between imidazole rings while in (**1**) and (**5**), the contact is between an imidazole ring and a benzyl ring. In particular, in (**1**) and (**5**) two mol­ecules centrosymmetrically related across the centre of symmetry at (0, ½, ½) are involved in π–π stacking in which the purine rings stack above the exocyclic phenyl ring, Table 11 ▸.

## Database survey   

A search made in the Cambridge Structural Database (Groom & Allen, 2014 ▸) revealed the existence of 11 deposited compounds containing the 2-thio-1-phenyl­ethanone scaffold (see supplementary Figure). Of those, only eight have a cyclic ring as substituent, the majority of these being heterocycles: MUCCUJ: 2-(1,3-benzoxazol-2-ylsulfan­yl)-1-phenyl­ethanone (Loghmani-Khouzani *et al.*, 2009*a* ▸); NENFAO: 3-(benzoyl­methyl­thio)-1,5-diphenyl-1*H*-1,2,4-triazole (Liu *et al.*, 2006 ▸); PUFGED: 2-(1,3-benzo­thia­zol-2-ylsulfan­yl)-1-phenyl­ethan­one (Loghmani-Khouzani *et al.*, 2009*b* ▸); IKAXOI: 6-cyclohexyl­methyl-5-ethyl-2-[(2-oxo-2-phenyl­eth­yl)sulfan­yl]pyrimidin-4(3*H*)-οne (Yan *et al.*, 2011 ▸); SILGAW: 2-(benzoylmethyl­sulfan­yl)-6-benzyl-5-iso­propyl­pyrimidin-4(3*H*)-one (Rao *et al.*, 2007 ▸); ETEWOP: 2-(benzoyl­methyl­sulphan­yl)-6-meth­oxy-1H-benzamide (Lynch & McLenaghan, 2004 ▸); XEBWEI: 2-(1,3-benzimidazolol-2-ylsulfan­yl)phenyl­ethan­one (Abdel-Aziz *et al.*, 2012 ▸); UGITUA: 2-[(4-meth­oxy­benz­yl)sulfan­yl]-1-phenyl­ethanone (Heravi *et al.*, 2009 ▸).

The *R*—S bond distances for these compounds are similar to those of the studied compounds and they assume a partial double-bond character with the exception of UGITUA where the S atom is bonded to a phenyl ring, suggesting a tendency for delocalization of the electron density through the sulfur atom when the ring has heteroatoms. The S—CH_2_ bond distances vary between 1.80 and 1.81 Å with exception of SILGAW (1.79 Å) and ETEWOP (1.82 Å). The supplementary figure also gives information about the distances of the –CH_2_– carbon atom to the best plane made up of the atoms of the heterocycles (CH_2_– distance). These values were computed in order to evaluate the degree of bending of the S—CH_2_ bond with respect to the main plane of the substituted rings. There are two main groups of compounds, one in which the distance is shorter than 0.3 Å and the other, which contains the CNH fragment in the heterocyclic ring, in which this distance is greater than 1.2 Å. As noted above, the *sp* ^3^ character of the β-carbon atom of the ethanone fragment may also direct the relative positions of the aceto­phenone residue out of the main plane constituted by the substituted heteroaromatic ring. This is the case for SILGAW and IKAXOI. Thus, despite the small sample size, there is a wide range of adopted conformations.

## Synthesis and crystallization   

The 6-mercaptopurine derivatives (**1**)–(**5**) were obtained in moderate yields by a two-step synthetic strategy. Firstly, 6-mercaptopurine was alkyl­ated using diverse monobromide aceto­phenone derivatives in DMF/potassium carbonate medium at room temperature (Lambertucci, *et al.* 2009 ▸). After thiol alkyl­ation, the purine nucleus was acyl­ated in position 9 with acetic anhydride in tri­ethyl­amine and anhydrous DMF for (**1**)–(**4**) under an argon atmosphere at room temperature (Masai, *et al.* 2002 ▸). All compounds were recrystallized from di­chloro­methane solution: 2-[(9-acetyl-9*H*-purin-6-yl)sulfan­yl]-1-(3-meth­oxy­phen­yl)ethan-1-one (**1**): overall yield: 48%; m.p. 432–435 K; 2-[(9-acetyl-9*H*-purin-6-yl)sulfan­yl]-1-(4-meth­oxy­phen­yl)ethan-1-one (**2**): overall yield: 17%; m.p. 460–463 K; 2-[(9-acetyl-9*H*-purin-6-yl)sulfan­yl]-1-(4-chloro­phen­yl)ethan-1-one (**3**): overall yield: 26%; m.p. 453–457 K; 2-[(9-acetyl-9*H*-purin-6-yl)sulfan­yl]-1-(4-bromo­phen­yl)ethan-1-one (**4**): overall yield: 10%; m.p. 449–451 K; 1-(3-meth­oxy­phen­yl)-2-[(9*H*-purin-6-yl)sulfan­yl]ethan-1-one (**5**): overall yield: 55%; m.p. 461–464 K.

## Refinement   

Crystal data, data collection and structure refinement details are summarized in Table 12 ▸. H atoms were treated as riding atoms with C—H(aromatic), 0.95 Å, with *U*_iso_ = 1.2*U*_eq_(C), C—H2(methyl­ene), 0.99 Å, with *U*_iso_ = 1.2*U*_eq_(C),*C*—H(meth­yl) 0.98 Å with *U*_iso_ = 1.5*U*_eq_(C) and in (**5**) only, N—H, 0.88 Å, with *U*_iso_ = 1.2*U*_eq_(C). The positions of the methyl groups were checked on a final difference map as was that of the N—H hydrogen atom in (**5**). In (**4**), the high difference map peaks were associated with the Br atom.

## Supplementary Material

Crystal structure: contains datablock(s) 1, 2, 3, 4, 5, global. DOI: 10.1107/S2056989016001833/hb7562sup1.cif

Structure factors: contains datablock(s) 1. DOI: 10.1107/S2056989016001833/hb75621sup2.hkl

Structure factors: contains datablock(s) 2. DOI: 10.1107/S2056989016001833/hb75622sup3.hkl

Structure factors: contains datablock(s) 3. DOI: 10.1107/S2056989016001833/hb75623sup4.hkl

Structure factors: contains datablock(s) 4. DOI: 10.1107/S2056989016001833/hb75624sup5.hkl

Structure factors: contains datablock(s) 5. DOI: 10.1107/S2056989016001833/hb75625sup6.hkl

Supporting information file. DOI: 10.1107/S2056989016001833/hb75621sup7.cml

Supporting information file. DOI: 10.1107/S2056989016001833/hb75622sup8.cml

Supporting information file. DOI: 10.1107/S2056989016001833/hb75623sup9.cml

Supporting information file. DOI: 10.1107/S2056989016001833/hb75624sup10.cml

Supporting information file. DOI: 10.1107/S2056989016001833/hb75625sup11.cml

CCDC references: 1450945, 1450944, 1450943, 1450942, 1450941

Additional supporting information:  crystallographic information; 3D view; checkCIF report

## Figures and Tables

**Figure 2:**
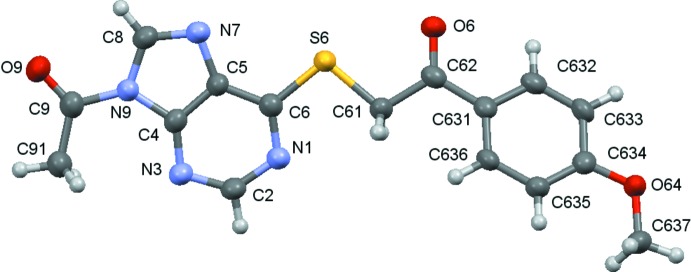
A view of the asymmetric unit of (**2**), with displacement ellipsoids are drawn at the 70% probability level.

**Figure 3:**
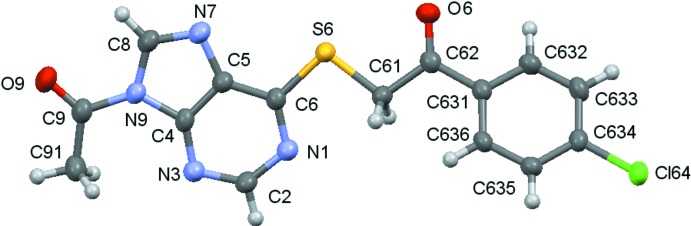
A view of the asymmetric unit of (**3**), with displacement ellipsoids are drawn at the 70% probability level.

**Figure 4:**
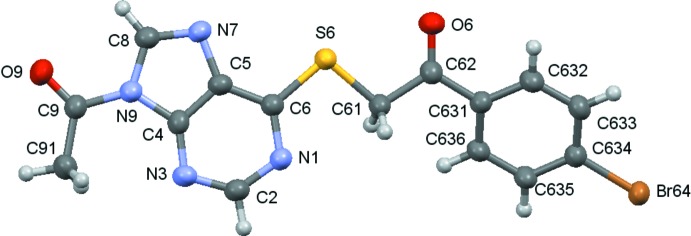
A view of the asymmetric unit of (**4**), with displacement ellipsoids are drawn at the 70% probability level.

**Figure 5:**
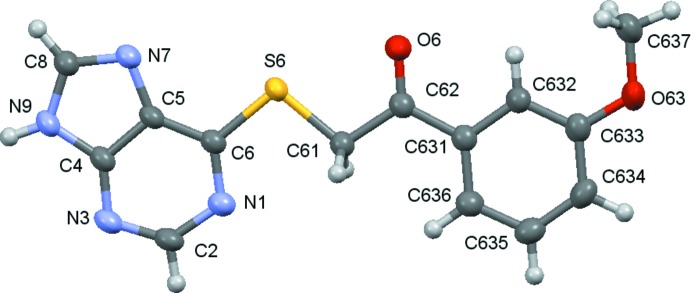
A view of the asymmetric unit of (**5**), with displacement ellipsoids are drawn at the 70% probability level.

**Table 1:** Selected geometric parameters (Å, °) for (**1**)

S6—C6	1.7438 (19)	C61—C62	1.520 (3)
S6—C61	1.8017 (18)	C62—C631	1.491 (3)
			
C6—S6—C61	100.76 (9)		
			
C6—S6—C61—C62	−178.05 (13)	S6—C61—C62—C631	−172.56 (14)
S6—C61—C62—O6	8.5 (2)		

**Table 2:** Selected geometric parameters (Å, °) for (**2**)

S6—C6	1.741 (3)	C61—C62	1.510 (4)
S6—C61	1.807 (3)	C62—C631	1.474 (3)
			
C6—S6—C61	100.88 (12)		
			
C6—S6—C61—C62	170.8 (2)	S6—C61—C62—C631	175.8 (2)
S6—C61—C62—O6	−7.7 (3)		

**Table 3:** Selected geometric parameters (Å, °) for (**3**)

S6—C6	1.7446 (18)	C61—C62	1.513 (2)
S6—C61	1.8038 (17)	C62—C631	1.493 (2)
			
C6—S6—C61	100.50 (8)		
			
C6—S6—C61—C62	177.77 (12)	S6—C61—C62—C631	177.32 (12)
S6—C61—C62—O6	−4.5 (2)		

**Table 4:** Selected geometric parameters (Å, °) for (**4**)

S6—C6	1.755 (3)	C61—C62	1.528 (4)
S6—C61	1.812 (3)	C62—C631	1.496 (4)
			
C6—S6—C61	100.33 (15)		
			
C6—S6—C61—C62	−177.8 (2)	S6—C61—C62—C631	−178.1 (2)
S6—C61—C62—O6	3.2 (4)		

**Table 5:** Selected geometric parameters (Å, °) for (**5**)

S6—C6	1.7477 (12)	C61—C62	1.5162 (16)
S6—C61	1.8109 (13)	C62—C631	1.4887 (17)
			
C6—S6—C61	100.77 (6)		
			
C6—S6—C61—C62	179.54 (8)	S6—C61—C62—C631	−175.65 (9)
S6—C61—C62—O6	5.56 (14)		

**Table 6:** Selected dihedral angles (°) θ_1_ is the dihedral angle between the mean planes of the purine and phenyl rings and the phenyl ring. θ_2_ is the dihedral angles between the mean planes of the purine ring and the plane defined by the S6/C61/C62/O6 atoms. θ_3_ is the dihedral angle between the mean planes of the phenyl ring and the plane defined by the S6/C61/C62/O6 atoms.

Compound	θ_1_°	θ_2_°	θ_3_°
(**1**)	2.95 (7)	8.45 (8)	5.87 (9)
(**2**)	38.89 (9)	17.05 (12)	22.72 (13)
(**3**)	38.67 (6)	14.23 (8)	27.82 (8)
(**4**)	37.11 (10)	13.58 (13)	26.82 (14)
(**5**)	4.74 (5)	5.30 (5)	3.42 (8)

The maximum deviations from the mean plane of the S–C–C–O bridging unit are for compounds (**1**)–(**5**) are 0.0457 (13), −0.041 (2), −0.023 (11), −0.017 (2) and 0.0302 (8) Å respectively. In all cases it is atom C42 which shows the maximum deviation.

**Table 7:** Hydrogen-bond geometry (Å, °) for (**2**)

*D*—H⋯*A*	*D*—H	H⋯*A*	*D*⋯*A*	*D*—H⋯*A*
C2—H2⋯O64^i^	0.95	2.48	3.375 (3)	157
C8—H8⋯O9^ii^	0.95	2.37	3.319 (3)	178
C61—H61*A*⋯O6^iii^	0.99	2.33	3.269 (3)	159

Symmetry codes: (i) 
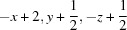
; (ii) 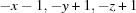; (iii) 
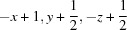
.

**Table 8:** Hydrogen-bond geometry (Å, °) for (**3**)

*D*—H⋯*A*	*D*—H	H⋯*A*	*D*⋯*A*	*D*—H⋯*A*
C8—H8⋯O9^i^	0.95	2.31	3.262 (2)	176
C61—H61*A*⋯O6^ii^	0.99	2.40	3.354 (2)	162

Symmetry codes: (i) 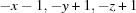; (ii) 
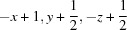
.

**Table 9:** Hydrogen-bond geometry (Å, °) for (**4**)

*D*—H⋯*A*	*D*—H	H⋯*A*	*D*⋯*A*	*D*—H⋯*A*
C8—H8⋯O9^i^	0.95	2.42	3.367 (4)	177
C61—H61*B*⋯O6^ii^	0.99	2.45	3.396 (4)	160

Symmetry codes: (i) 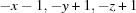; (ii) 
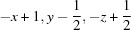
.

**Table 10:** Hydrogen-bond geometry (Å, °) for (**5**)

*D*—H⋯*A*	*D*—H	H⋯*A*	*D*⋯*A*	*D*—H⋯*A*
N9—H9⋯N7^i^	0.88	1.90	2.7715 (14)	171

Symmetry code: (i) 
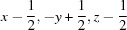
.

**Table 11:** Selected π–π contacts (Å, °) *CgI*(*J*) is plane *I*(*J*); *Cg*⋯*Cg* is the distance between ring centroids; α is the dihedral angle between planes *I* and *J*; *CgI*_perp_ is the perpendicular distance of *Cg*(*I*) on ring *J*; *CgJ*_perp_ is the perpendicular distance of *Cg*(J) on ring *I*; Slippage is the distance between *Cg*(*I*) and the perpendicular projection of *Cg*(*J*) on ring *I*. Plane 1 is through the imadazole ring, plane 2 the pyrimidine ring and plane 3 the exocyclic benzene ring.

Compound	*CgI*	*CgJ*	*Cg*⋯*Cg*	α	*CgI*_perp_	*CgJ*_perp_	Slippage
(**1**)	*Cg*1	*Cg*3(−*x*, 1 − *y*, 1 − *z*)	3.6923 (14)	2.62 (12)	3.4547 (9)	−3.3985 (9)	
	*Cg*2	*Cg*3(−*x*, 1 − *y*, 1 − *z*)	3.6019 (12)	3.26 (11)	−3.3477 (9)	−3.4071 (9)	
(**2**)	*Cg*1	*Cg*1(−*x*, 1 − *y*, −*z*)	3.8561 (16)	0.00 (15)	3.3156 (11)	3.3156 (11)	1.969
	*Cg*2	*Cg*3(1 − *x*, 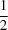 + *y*, 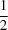 − *z*)	3.8270 (16)	0.80 (12)	−3.2463 (10)	−3.2391 (11)	
(**3**)	*Cg*1	*Cg*1(−*x*, 1 − *y*, −*z*)	3.7799 (11)	0	3.2016 (7)	3.2016 (7)	2.009
	*Cg*2	*Cg*3(1 − *x*, 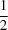 + *y*, 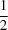 − *z*)	4.0620 (10)	6.70 (8)	−3.4438 (7)	−3.1708 (7)	
(**4**)	*Cg*1	*Cg*1(1 − *x*, 1 − *y*, 1 − *z*)	3.8319 (18)	0.04 (18)	3.1987 (13)	3.1987 (13)	2.110
	*Cg*2	*Cg*3(1 − *x*, 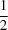 + *y*, 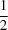 − *z*)	4.1601 (18)	6.27 (15)	−3.4328 (12)	−3.1701 (13)	
(**5**)	*Cg*1	*Cg*3(−*x*, 1 − *y*, 1 − *z*)	3.6359 (8)	5.35 (7)	−3.4757 (5)	−3.4162 (5)	
	*Cg*2	*Cg*3(−*x*, 1 − *y*, 1 − *z*)	3.5204 (8)	4.43 (6)	−3.3669 (5)	−3.4160 (5)	

**Table:** 

	(**1**)	(**2**)	(**3**)
Crystal data			
Chemical formula	C_16_H_14_N_4_O_3_S	C_16_H_14_N_4_O_3_S	C_15_H_11_ClN_4_O_2_S
*M*_r_	342.37	342.37	346.79
Crystal system, space group	Monoclinic, *P*2_1_/*n*	Monoclinic, *P*2_1_/*c*	Monoclinic, *P*2_1_/*c*
Temperature (K)	100	100	100
*a*, *b*, *c* (Å)	7.6343 (5), 26.2356 (18), 8.1332 (5)	5.9920 (3), 9.9795 (5), 24.9907 (13)	5.9900 (4), 9.9169 (7), 24.3238 (17)
β (°)	112.725 (2)	95.977 (5)	96.072 (2)
*V* (Å^3^)	1502.54 (17)	1486.25 (13)	1436.78 (17)
*Z*	4	4	4
Radiation type	Mo *K*α	Mo *K*α	Mo *K*α
μ (mm^−1^)	0.24	0.24	0.43
Crystal size (mm)	0.17 × 0.07 × 0.01	0.05 × 0.04 × 0.01	0.13 × 0.06 × 0.01

Data collection			
Diffractometer	Rigaku AFC12 (Right)	Rigaku AFC12 (Right)	Rigaku AFC12 (Right)
Absorption correction	Multi-scan (*CrystalClear-SM Expert*; Rigaku, 20112)	Multi-scan (*CrysAlis PRO*; Agilent, 2014 ▸)	Multi-scan *CrystalClear-SM Expert* (Rigaku, 20112)
*T*_min_, *T*_max_	0, 1.000	0.439, 1.000	0.809, 1.000
No. of measured, independent and observed [*I* > 2σ(*I*)] reflections	20144, 3450, 2817	15437, 2619, 1852	18353, 3291, 2677
*R*_int_	0.089	0.106	0.050
(sin θ/λ)_max_ (Å^−1^)	0.649	0.595	0.651

Refinement			
*R*[*F* ^2^ > 2σ(*F* ^2^)], *wR*(*F* ^2^), *S*	0.053, 0.148, 1.05	0.048, 0.116, 1.02	0.035, 0.092, 1.02
No. of reflections	3450	2619	3291
No. of parameters	219	219	209
H-atom treatment	H-atom parameters constrained	H-atom parameters constrained	H-atom parameters constrained
Δρ_max_, Δρ_min_ (e Å^−3^)	0.81, −0.51	0.30, −0.36	0.34, −0.22

**Table:** 

	(**4**)	(**5**)
Crystal data		
Chemical formula	C_15_H_11_BrN_4_O_2_S	C_14_H_12_N_4_O_2_S
*M*_r_	391.25	300.34
Crystal system, space group	Monoclinic, *P*2_1_/*c*	Monoclinic, *P*2_1_/*n*
Temperature (K)	100	100
*a*, *b*, *c* (Å)	6.0705 (4), 10.0668 (7), 24.3492 (17)	7.6683 (5), 21.8004 (15), 8.4131 (5)
β (°)	96.580 (2)	107.507 (2)
*V* (Å^3^)	1478.19 (18)	1341.29 (15)
*Z*	4	4
Radiation type	Mo *K*α	Mo *K*α
μ (mm^−1^)	2.94	0.25
Crystal size (mm)	0.15 × 0.10 × 0.02	0.17 × 0.12 × 0.07

Data collection		
Diffractometer	Rigaku AFC12 (Right)	Rigaku AFC12 (Right)
Absorption correction	Multi-scan *CrystalClear-SM Expert* (Rigaku, 20112)	Multi-scan (*CrystalClear-SM Expert*; Rigaku, 2012 ▸)
*T*_min_, *T*_max_	0.658, 1.000	0.724, 1.000
No. of measured, independent and observed [*I* > 2σ(*I*)] reflections	18171, 3346, 2944	17441, 3063, 2799
*R*_int_	0.064	0.060
(sin θ/λ)_max_ (Å^−1^)	0.649	0.649

Refinement		
*R*[*F* ^2^ > 2σ(*F* ^2^)], *wR*(*F* ^2^), *S*	0.056, 0.153, 1.06	0.033, 0.093, 1.03
No. of reflections	3346	3063
No. of parameters	209	191
H-atom treatment	H-atom parameters constrained	H-atom parameters constrained
Δρ_max_, Δρ_min_ (e Å^−3^)	2.91, −0.92	0.30, −0.37

Computer programs: *CrystalClear-SM Expert* (Rigaku, 2012 ▸), *CrysAlis PRO* (Agilent, 2014 ▸), *SHELXT* (Sheldrick, 2015*a* ▸), *ShelXle* (Hübschle *et al.*, 2011 ▸), *SHELXL2014* (Sheldrick, 2015*b* ▸), *OSCAIL* (McArdle *et al.*, 2004 ▸), *Mercury* (Macrae *et al.*, 2006 ▸), and *PLATON* (Spek, 2009 ▸).

## supplementary crystallographic information

### (1) 2-[(9-Acetyl-9H-purin-6-yl)sulfanyl]-1-(3-methoxyphenyl)ethan-1-one Crystal data

**Table:**

C_16_H_14_N_4_O_3_S	*F*(000) = 712
*M**_r_* = 342.37	*D*_x_ = 1.513 Mg m^−^^3^
Monoclinic, *P*2_1_/*n*	Mo *K*α radiation, λ = 0.71075 Å
*a* = 7.6343 (5) Å	Cell parameters from 18327 reflections
*b* = 26.2356 (18) Å	θ = 2.7–27.5°
*c* = 8.1332 (5) Å	µ = 0.24 mm^−^^1^
β = 112.725 (2)°	*T* = 100 K
*V* = 1502.54 (17) Å^3^	Plate, orange
*Z* = 4	0.17 × 0.07 × 0.01 mm

### (1) 2-[(9-Acetyl-9H-purin-6-yl)sulfanyl]-1-(3-methoxyphenyl)ethan-1-one Data collection

**Table:**

Rigaku AFC12 (Right) diffractometer	3450 independent reflections
Radiation source: Rotating Anode	2817 reflections with *I* > 2σ(*I*)
Detector resolution: 28.5714 pixels mm^-1^	*R*_int_ = 0.089
profile data from ω–scans	θ_max_ = 27.5°, θ_min_ = 2.8°
Absorption correction: multi-scan (*CrystalClear-SM Expert*; Rigaku, 20112)	*h* = −9→9
*T*_min_ = 0, *T*_max_ = 1.000	*k* = −33→34
20144 measured reflections	*l* = −10→10

### (1) 2-[(9-Acetyl-9H-purin-6-yl)sulfanyl]-1-(3-methoxyphenyl)ethan-1-one Refinement

**Table:**

Refinement on *F*^2^	0 restraints
Least-squares matrix: full	Hydrogen site location: inferred from neighbouring sites
*R*[*F*^2^ > 2σ(*F*^2^)] = 0.053	H-atom parameters constrained
*wR*(*F*^2^) = 0.148	*w* = 1/[σ^2^(*F*_o_^2^) + (0.0878*P*)^2^ + 0.318*P*] where *P* = (*F*_o_^2^ + 2*F*_c_^2^)/3
*S* = 1.05	(Δ/σ)_max_ = 0.002
3450 reflections	Δρ_max_ = 0.81 e Å^−^^3^
219 parameters	Δρ_min_ = −0.51 e Å^−^^3^

### (1) 2-[(9-Acetyl-9H-purin-6-yl)sulfanyl]-1-(3-methoxyphenyl)ethan-1-one Special details

**Table:**

Geometry. All e.s.d.'s (except the e.s.d. in the dihedral angle between two l.s. planes) are estimated using the full covariance matrix. The cell e.s.d.'s are taken into account individually in the estimation of e.s.d.'s in distances, angles and torsion angles; correlations between e.s.d.'s in cell parameters are only used when they are defined by crystal symmetry. An approximate (isotropic) treatment of cell e.s.d.'s is used for estimating e.s.d.'s involving l.s. planes.

### (1) 2-[(9-Acetyl-9H-purin-6-yl)sulfanyl]-1-(3-methoxyphenyl)ethan-1-one Fractional atomic coordinates and isotropic or equivalent isotropic displacement parameters (Å^2^)

**Table:**

	*x*	*y*	*z*	*U*_iso_*/*U*_eq_	
S6	0.32576 (7)	0.51901 (2)	0.60919 (6)	0.03400 (17)	
O6	0.24518 (19)	0.61509 (5)	0.4576 (2)	0.0421 (4)	
O9	0.8227 (2)	0.31676 (6)	1.1619 (2)	0.0427 (4)	
O63	−0.20860 (19)	0.72411 (5)	−0.05304 (18)	0.0376 (3)	
N1	0.2055 (2)	0.42325 (6)	0.4964 (2)	0.0331 (4)	
N3	0.3481 (2)	0.34590 (6)	0.6538 (2)	0.0323 (4)	
N7	0.6212 (2)	0.44997 (6)	0.9142 (2)	0.0340 (4)	
N9	0.6216 (2)	0.36406 (6)	0.9348 (2)	0.0325 (4)	
C2	0.2207 (3)	0.37227 (7)	0.5197 (3)	0.0342 (4)	
H2	0.1291	0.3525	0.4292	0.041*	
C4	0.4710 (3)	0.37649 (7)	0.7754 (3)	0.0309 (4)	
C5	0.4740 (3)	0.42955 (7)	0.7676 (2)	0.0312 (4)	
C6	0.3330 (3)	0.45263 (7)	0.6203 (2)	0.0308 (4)	
C8	0.7025 (3)	0.41060 (7)	1.0084 (3)	0.0343 (4)	
H8	0.8086	0.4132	1.1185	0.041*	
C9	0.6911 (3)	0.31650 (7)	1.0206 (3)	0.0344 (4)	
C61	0.1558 (3)	0.52851 (7)	0.3849 (2)	0.0321 (4)	
H61A	0.1996	0.5115	0.2989	0.039*	
H61B	0.0313	0.5139	0.3711	0.039*	
C62	0.1376 (3)	0.58558 (7)	0.3504 (3)	0.0322 (4)	
C91	0.5932 (3)	0.26954 (7)	0.9240 (3)	0.0396 (5)	
H91A	0.6527	0.2394	0.9945	0.059*	
H91B	0.6040	0.2678	0.8080	0.059*	
H91C	0.4588	0.2707	0.9064	0.059*	
C631	−0.0135 (2)	0.60328 (7)	0.1806 (2)	0.0312 (4)	
C632	−0.0336 (3)	0.65606 (7)	0.1486 (2)	0.0317 (4)	
H632	0.0474	0.6794	0.2332	0.038*	
C633	−0.1734 (3)	0.67364 (7)	−0.0084 (3)	0.0328 (4)	
C634	−0.2922 (3)	0.63923 (8)	−0.1321 (3)	0.0361 (4)	
H634	−0.3883	0.6514	−0.2390	0.043*	
C635	−0.2701 (3)	0.58745 (8)	−0.0994 (3)	0.0373 (4)	
H635	−0.3502	0.5642	−0.1849	0.045*	
C636	−0.1321 (3)	0.56914 (7)	0.0572 (3)	0.0352 (4)	
H636	−0.1188	0.5335	0.0798	0.042*	
C637	−0.0942 (3)	0.76081 (7)	0.0721 (3)	0.0396 (5)	
H63A	0.0390	0.7563	0.0885	0.059*	
H63B	−0.1070	0.7561	0.1864	0.059*	
H63C	−0.1362	0.7952	0.0271	0.059*	

### (1) 2-[(9-Acetyl-9H-purin-6-yl)sulfanyl]-1-(3-methoxyphenyl)ethan-1-one Atomic displacement parameters (Å^2^)

**Table:**

	*U*^11^	*U*^22^	*U*^33^	*U*^12^	*U*^13^	*U*^23^
S6	0.0278 (3)	0.0304 (3)	0.0354 (3)	0.00002 (17)	0.0030 (2)	−0.00035 (17)
O6	0.0327 (7)	0.0341 (7)	0.0442 (8)	−0.0027 (6)	−0.0019 (6)	0.0004 (6)
O9	0.0351 (8)	0.0417 (8)	0.0422 (8)	0.0012 (6)	0.0048 (7)	0.0043 (6)
O63	0.0322 (7)	0.0346 (7)	0.0376 (7)	0.0025 (5)	0.0042 (6)	0.0021 (5)
N1	0.0283 (8)	0.0332 (8)	0.0347 (8)	−0.0007 (6)	0.0088 (6)	−0.0002 (6)
N3	0.0280 (8)	0.0333 (8)	0.0337 (8)	−0.0013 (6)	0.0097 (7)	−0.0008 (6)
N7	0.0244 (8)	0.0361 (8)	0.0368 (9)	−0.0011 (6)	0.0066 (7)	−0.0020 (6)
N9	0.0241 (8)	0.0344 (8)	0.0358 (8)	0.0015 (6)	0.0080 (7)	0.0013 (6)
C2	0.0303 (9)	0.0347 (9)	0.0341 (10)	−0.0021 (7)	0.0087 (8)	−0.0023 (7)
C4	0.0236 (8)	0.0342 (9)	0.0336 (9)	0.0014 (7)	0.0096 (7)	0.0008 (7)
C5	0.0240 (8)	0.0324 (9)	0.0341 (9)	0.0000 (7)	0.0080 (7)	−0.0005 (7)
C6	0.0261 (9)	0.0309 (9)	0.0342 (9)	0.0002 (7)	0.0102 (8)	−0.0010 (7)
C8	0.0246 (8)	0.0378 (10)	0.0366 (10)	−0.0017 (7)	0.0076 (8)	−0.0022 (8)
C9	0.0280 (9)	0.0367 (10)	0.0389 (10)	0.0029 (7)	0.0133 (8)	0.0033 (8)
C61	0.0246 (9)	0.0324 (9)	0.0331 (10)	−0.0005 (7)	0.0041 (7)	0.0007 (7)
C62	0.0245 (8)	0.0342 (9)	0.0344 (9)	−0.0009 (7)	0.0077 (7)	−0.0003 (7)
C91	0.0345 (10)	0.0368 (10)	0.0442 (11)	0.0011 (8)	0.0115 (9)	0.0006 (8)
C631	0.0231 (8)	0.0362 (9)	0.0329 (9)	0.0000 (7)	0.0092 (7)	0.0010 (7)
C632	0.0249 (9)	0.0343 (9)	0.0326 (9)	−0.0007 (7)	0.0076 (7)	−0.0003 (7)
C633	0.0260 (9)	0.0360 (9)	0.0351 (10)	0.0032 (7)	0.0103 (8)	0.0028 (7)
C634	0.0266 (9)	0.0455 (11)	0.0318 (10)	0.0024 (8)	0.0065 (8)	0.0014 (8)
C635	0.0290 (9)	0.0423 (10)	0.0357 (10)	−0.0043 (8)	0.0072 (8)	−0.0058 (8)
C636	0.0301 (9)	0.0349 (9)	0.0374 (10)	−0.0014 (7)	0.0096 (8)	−0.0007 (7)
C637	0.0346 (10)	0.0351 (10)	0.0425 (11)	0.0010 (8)	0.0075 (9)	0.0011 (8)

### (1) 2-[(9-Acetyl-9H-purin-6-yl)sulfanyl]-1-(3-methoxyphenyl)ethan-1-one Geometric parameters (Å, º)

**Table:**

S6—C6	1.7438 (19)	C61—C62	1.520 (3)
S6—C61	1.8017 (18)	C61—H61A	0.9900
O6—C62	1.217 (2)	C61—H61B	0.9900
O9—C9	1.199 (2)	C62—C631	1.491 (3)
O63—C633	1.372 (2)	C91—H91A	0.9800
O63—C637	1.428 (2)	C91—H91B	0.9800
N1—C6	1.341 (2)	C91—H91C	0.9800
N1—C2	1.350 (2)	C631—C636	1.388 (3)
N3—C4	1.336 (2)	C631—C632	1.407 (3)
N3—C2	1.340 (2)	C632—C633	1.389 (3)
N7—C8	1.293 (2)	C632—H632	0.9500
N7—C5	1.392 (2)	C633—C634	1.395 (3)
N9—C8	1.395 (2)	C634—C635	1.382 (3)
N9—C4	1.399 (2)	C634—H634	0.9500
N9—C9	1.428 (2)	C635—C636	1.388 (3)
C2—H2	0.9500	C635—H635	0.9500
C4—C5	1.394 (3)	C636—H636	0.9500
C5—C6	1.401 (2)	C637—H63A	0.9800
C8—H8	0.9500	C637—H63B	0.9800
C9—C91	1.496 (3)	C637—H63C	0.9800
			
C6—S6—C61	100.76 (9)	O6—C62—C61	120.45 (17)
C633—O63—C637	117.31 (15)	C631—C62—C61	117.42 (15)
C6—N1—C2	117.76 (16)	C9—C91—H91A	109.5
C4—N3—C2	111.95 (16)	C9—C91—H91B	109.5
C8—N7—C5	104.13 (16)	H91A—C91—H91B	109.5
C8—N9—C4	105.22 (15)	C9—C91—H91C	109.5
C8—N9—C9	122.41 (16)	H91A—C91—H91C	109.5
C4—N9—C9	132.36 (16)	H91B—C91—H91C	109.5
N3—C2—N1	128.47 (17)	C636—C631—C632	120.49 (17)
N3—C2—H2	115.8	C636—C631—C62	121.58 (17)
N1—C2—H2	115.8	C632—C631—C62	117.92 (16)
N3—C4—C5	125.80 (17)	C633—C632—C631	119.17 (17)
N3—C4—N9	129.53 (17)	C633—C632—H632	120.4
C5—C4—N9	104.66 (16)	C631—C632—H632	120.4
N7—C5—C4	111.55 (16)	O63—C633—C632	124.49 (17)
N7—C5—C6	131.72 (17)	O63—C633—C634	115.30 (16)
C4—C5—C6	116.73 (16)	C632—C633—C634	120.20 (17)
N1—C6—C5	119.27 (16)	C635—C634—C633	120.01 (18)
N1—C6—S6	122.38 (14)	C635—C634—H634	120.0
C5—C6—S6	118.31 (14)	C633—C634—H634	120.0
N7—C8—N9	114.43 (17)	C634—C635—C636	120.66 (18)
N7—C8—H8	122.8	C634—C635—H635	119.7
N9—C8—H8	122.8	C636—C635—H635	119.7
O9—C9—N9	118.60 (18)	C631—C636—C635	119.46 (18)
O9—C9—C91	124.84 (18)	C631—C636—H636	120.3
N9—C9—C91	116.55 (17)	C635—C636—H636	120.3
C62—C61—S6	107.55 (12)	O63—C637—H63A	109.5
C62—C61—H61A	110.2	O63—C637—H63B	109.5
S6—C61—H61A	110.2	H63A—C637—H63B	109.5
C62—C61—H61B	110.2	O63—C637—H63C	109.5
S6—C61—H61B	110.2	H63A—C637—H63C	109.5
H61A—C61—H61B	108.5	H63B—C637—H63C	109.5
O6—C62—C631	122.13 (17)		
			
C4—N3—C2—N1	0.3 (3)	C9—N9—C8—N7	−179.35 (18)
C6—N1—C2—N3	0.6 (3)	C8—N9—C9—O9	0.7 (3)
C2—N3—C4—C5	−1.2 (3)	C4—N9—C9—O9	−178.2 (2)
C2—N3—C4—N9	178.84 (19)	C8—N9—C9—C91	−178.60 (18)
C8—N9—C4—N3	179.7 (2)	C4—N9—C9—C91	2.5 (3)
C9—N9—C4—N3	−1.3 (4)	C6—S6—C61—C62	−178.05 (13)
C8—N9—C4—C5	−0.3 (2)	S6—C61—C62—O6	8.5 (2)
C9—N9—C4—C5	178.73 (19)	S6—C61—C62—C631	−172.56 (14)
C8—N7—C5—C4	−0.8 (2)	O6—C62—C631—C636	178.23 (19)
C8—N7—C5—C6	178.5 (2)	C61—C62—C631—C636	−0.7 (3)
N3—C4—C5—N7	−179.29 (18)	O6—C62—C631—C632	−2.1 (3)
N9—C4—C5—N7	0.7 (2)	C61—C62—C631—C632	179.03 (17)
N3—C4—C5—C6	1.3 (3)	C636—C631—C632—C633	−0.2 (3)
N9—C4—C5—C6	−178.75 (16)	C62—C631—C632—C633	−179.87 (17)
C2—N1—C6—C5	−0.5 (3)	C637—O63—C633—C632	−0.7 (3)
C2—N1—C6—S6	−178.35 (15)	C637—O63—C633—C634	178.19 (18)
N7—C5—C6—N1	−179.6 (2)	C631—C632—C633—O63	178.91 (17)
C4—C5—C6—N1	−0.3 (3)	C631—C632—C633—C634	0.1 (3)
N7—C5—C6—S6	−1.6 (3)	O63—C633—C634—C635	−179.39 (18)
C4—C5—C6—S6	177.61 (14)	C632—C633—C634—C635	−0.5 (3)
C61—S6—C6—N1	−12.10 (18)	C633—C634—C635—C636	0.9 (3)
C61—S6—C6—C5	170.02 (16)	C632—C631—C636—C635	0.6 (3)
C5—N7—C8—N9	0.6 (2)	C62—C631—C636—C635	−179.72 (18)
C4—N9—C8—N7	−0.2 (2)	C634—C635—C636—C631	−1.0 (3)

### (2) 2-[(9-Acetyl-9H-purin-6-yl)sulfanyl]-1-(4-methoxyphenyl)ethan-1-one Crystal data

**Table:**

C_16_H_14_N_4_O_3_S	*F*(000) = 712
*M**_r_* = 342.37	*D*_x_ = 1.530 Mg m^−^^3^
Monoclinic, *P*2_1_/*c*	Mo *K*α radiation, λ = 0.71073 Å
*a* = 5.9920 (3) Å	Cell parameters from 5825 reflections
*b* = 9.9795 (5) Å	θ = 2.2–25.0°
*c* = 24.9907 (13) Å	µ = 0.24 mm^−^^1^
β = 95.977 (5)°	*T* = 100 K
*V* = 1486.25 (13) Å^3^	Plate, colourless
*Z* = 4	0.05 × 0.04 × 0.01 mm

### (2) 2-[(9-Acetyl-9H-purin-6-yl)sulfanyl]-1-(4-methoxyphenyl)ethan-1-one Data collection

**Table:**

Rigaku AFC12 (Right) diffractometer	2619 independent reflections
Radiation source: Rotating Anode, Rotating Anode	1852 reflections with *I* > 2σ(*I*)
Confocal mirrors, HF Varimax monochromator	*R*_int_ = 0.106
Detector resolution: 28.5714 pixels mm^-1^	θ_max_ = 25.0°, θ_min_ = 2.2°
profile data from ω–scans	*h* = −7→6
Absorption correction: multi-scan (*CrysAlis PRO*; Agilent, 2014)	*k* = −11→11
*T*_min_ = 0.439, *T*_max_ = 1.000	*l* = −29→29
15437 measured reflections	

### (2) 2-[(9-Acetyl-9H-purin-6-yl)sulfanyl]-1-(4-methoxyphenyl)ethan-1-one Refinement

**Table:**

Refinement on *F*^2^	0 restraints
Least-squares matrix: full	Hydrogen site location: inferred from neighbouring sites
*R*[*F*^2^ > 2σ(*F*^2^)] = 0.048	H-atom parameters constrained
*wR*(*F*^2^) = 0.116	*w* = 1/[σ^2^(*F*_o_^2^) + (0.0492*P*)^2^ + 0.3956*P*] where *P* = (*F*_o_^2^ + 2*F*_c_^2^)/3
*S* = 1.02	(Δ/σ)_max_ < 0.001
2619 reflections	Δρ_max_ = 0.30 e Å^−^^3^
219 parameters	Δρ_min_ = −0.36 e Å^−^^3^

### (2) 2-[(9-Acetyl-9H-purin-6-yl)sulfanyl]-1-(4-methoxyphenyl)ethan-1-one Special details

**Table:**

Geometry. All e.s.d.'s (except the e.s.d. in the dihedral angle between two l.s. planes) are estimated using the full covariance matrix. The cell e.s.d.'s are taken into account individually in the estimation of e.s.d.'s in distances, angles and torsion angles; correlations between e.s.d.'s in cell parameters are only used when they are defined by crystal symmetry. An approximate (isotropic) treatment of cell e.s.d.'s is used for estimating e.s.d.'s involving l.s. planes.

### (2) 2-[(9-Acetyl-9H-purin-6-yl)sulfanyl]-1-(4-methoxyphenyl)ethan-1-one Fractional atomic coordinates and isotropic or equivalent isotropic displacement parameters (Å^2^)

**Table:**

	*x*	*y*	*z*	*U*_iso_*/*U*_eq_	
S6	0.34226 (12)	0.45424 (7)	0.33305 (3)	0.0300 (2)	
O6	0.6113 (3)	0.30045 (19)	0.27249 (7)	0.0315 (5)	
O9	−0.4124 (3)	0.6642 (2)	0.51385 (8)	0.0386 (6)	
O64	1.2947 (3)	0.54050 (18)	0.12232 (7)	0.0331 (5)	
N1	0.3919 (4)	0.7064 (2)	0.37258 (9)	0.0274 (6)	
N3	0.1587 (4)	0.8075 (2)	0.43517 (9)	0.0282 (6)	
N7	−0.0486 (4)	0.4737 (2)	0.41015 (9)	0.0295 (6)	
N9	−0.1340 (4)	0.6524 (2)	0.45982 (8)	0.0268 (6)	
C2	0.3246 (5)	0.8061 (3)	0.40336 (10)	0.0286 (7)	
H2	0.4069	0.8873	0.4023	0.034*	
C4	0.0500 (4)	0.6901 (3)	0.43323 (10)	0.0260 (7)	
C5	0.0983 (4)	0.5794 (3)	0.40346 (10)	0.0251 (6)	
C6	0.2774 (5)	0.5909 (3)	0.37190 (11)	0.0264 (7)	
C8	−0.1807 (5)	0.5208 (3)	0.44369 (11)	0.0293 (7)	
H8	−0.2989	0.4699	0.4561	0.035*	
C9	−0.2672 (5)	0.7242 (3)	0.49399 (11)	0.0316 (7)	
C61	0.5465 (5)	0.5280 (3)	0.29356 (11)	0.0296 (7)	
H61A	0.4725	0.5938	0.2679	0.036*	
H61B	0.6632	0.5752	0.3174	0.036*	
C62	0.6521 (5)	0.4179 (3)	0.26328 (10)	0.0265 (7)	
C91	−0.2175 (5)	0.8699 (3)	0.50110 (11)	0.0366 (8)	
H91A	−0.2266	0.9134	0.4658	0.055*	
H91B	−0.0662	0.8814	0.5195	0.055*	
H91C	−0.3271	0.9106	0.5226	0.055*	
C631	0.8133 (4)	0.4560 (3)	0.22523 (10)	0.0257 (6)	
C632	0.9745 (5)	0.3617 (3)	0.21303 (10)	0.0279 (7)	
H632	0.9736	0.2748	0.2285	0.033*	
C633	1.1329 (5)	0.3927 (3)	0.17933 (10)	0.0278 (7)	
H633	1.2425	0.3284	0.1719	0.033*	
C634	1.1318 (5)	0.5197 (3)	0.15592 (11)	0.0279 (7)	
C635	0.9733 (4)	0.6143 (3)	0.16630 (10)	0.0276 (7)	
H635	0.9713	0.6998	0.1496	0.033*	
C636	0.8169 (4)	0.5822 (3)	0.20161 (10)	0.0264 (7)	
H636	0.7105	0.6477	0.2098	0.032*	
C637	1.3246 (5)	0.6732 (3)	0.10294 (12)	0.0362 (8)	
H63A	1.4617	0.6770	0.0848	0.054*	
H63B	1.3370	0.7360	0.1332	0.054*	
H63C	1.1955	0.6977	0.0775	0.054*	

### (2) 2-[(9-Acetyl-9H-purin-6-yl)sulfanyl]-1-(4-methoxyphenyl)ethan-1-one Atomic displacement parameters (Å^2^)

**Table:**

	*U*^11^	*U*^22^	*U*^33^	*U*^12^	*U*^13^	*U*^23^
S6	0.0341 (4)	0.0194 (4)	0.0394 (4)	−0.0023 (3)	0.0176 (3)	−0.0022 (3)
O6	0.0380 (12)	0.0199 (12)	0.0388 (11)	−0.0023 (9)	0.0149 (10)	0.0002 (9)
O9	0.0413 (12)	0.0305 (13)	0.0479 (13)	−0.0035 (10)	0.0236 (11)	−0.0003 (10)
O64	0.0377 (12)	0.0206 (12)	0.0448 (12)	0.0004 (9)	0.0228 (10)	0.0014 (9)
N1	0.0290 (13)	0.0206 (14)	0.0340 (13)	−0.0009 (11)	0.0105 (11)	0.0005 (11)
N3	0.0297 (13)	0.0230 (14)	0.0333 (13)	−0.0040 (11)	0.0107 (11)	−0.0009 (10)
N7	0.0326 (14)	0.0206 (14)	0.0372 (13)	−0.0017 (11)	0.0128 (12)	0.0014 (11)
N9	0.0278 (13)	0.0205 (14)	0.0340 (13)	0.0006 (10)	0.0119 (11)	0.0012 (11)
C2	0.0292 (16)	0.0226 (17)	0.0352 (16)	−0.0051 (13)	0.0087 (14)	−0.0006 (13)
C4	0.0273 (16)	0.0251 (17)	0.0267 (15)	−0.0001 (13)	0.0081 (13)	0.0030 (12)
C5	0.0237 (14)	0.0217 (17)	0.0311 (15)	−0.0002 (12)	0.0091 (13)	0.0011 (12)
C6	0.0293 (15)	0.0223 (17)	0.0286 (15)	0.0013 (13)	0.0068 (13)	0.0007 (12)
C8	0.0279 (15)	0.0247 (18)	0.0366 (16)	−0.0026 (13)	0.0101 (14)	0.0031 (13)
C9	0.0320 (16)	0.0287 (18)	0.0358 (16)	0.0032 (14)	0.0116 (14)	0.0017 (13)
C61	0.0370 (17)	0.0208 (17)	0.0336 (16)	0.0006 (13)	0.0160 (14)	0.0024 (12)
C62	0.0305 (16)	0.0195 (17)	0.0296 (15)	0.0010 (13)	0.0030 (13)	−0.0020 (12)
C91	0.0425 (18)	0.0283 (18)	0.0421 (17)	−0.0003 (14)	0.0190 (16)	−0.0020 (14)
C631	0.0272 (15)	0.0210 (16)	0.0298 (15)	−0.0028 (12)	0.0066 (13)	−0.0049 (12)
C632	0.0360 (17)	0.0178 (16)	0.0303 (15)	−0.0002 (13)	0.0054 (14)	−0.0028 (12)
C633	0.0303 (15)	0.0197 (16)	0.0344 (15)	0.0056 (12)	0.0083 (14)	−0.0031 (13)
C634	0.0310 (16)	0.0236 (17)	0.0302 (15)	−0.0045 (13)	0.0090 (13)	−0.0033 (12)
C635	0.0316 (16)	0.0188 (16)	0.0338 (16)	−0.0006 (13)	0.0105 (14)	0.0011 (12)
C636	0.0271 (15)	0.0170 (16)	0.0362 (16)	0.0009 (12)	0.0091 (13)	−0.0033 (12)
C637	0.0448 (19)	0.0243 (17)	0.0430 (18)	−0.0049 (14)	0.0218 (16)	0.0008 (14)

### (2) 2-[(9-Acetyl-9H-purin-6-yl)sulfanyl]-1-(4-methoxyphenyl)ethan-1-one Geometric parameters (Å, º)

**Table:**

S6—C6	1.741 (3)	C61—C62	1.510 (4)
S6—C61	1.807 (3)	C61—H61A	0.9900
O6—C62	1.224 (3)	C61—H61B	0.9900
O9—C9	1.206 (3)	C62—C631	1.474 (3)
O64—C634	1.368 (3)	C91—H91A	0.9800
O64—C637	1.428 (3)	C91—H91B	0.9800
N1—C6	1.340 (3)	C91—H91C	0.9800
N1—C2	1.345 (3)	C631—C636	1.393 (4)
N3—C2	1.336 (3)	C631—C632	1.404 (4)
N3—C4	1.339 (3)	C632—C633	1.368 (4)
N7—C8	1.299 (3)	C632—H632	0.9500
N7—C5	1.395 (3)	C633—C634	1.395 (4)
N9—C8	1.394 (3)	C633—H633	0.9500
N9—C4	1.396 (3)	C634—C635	1.383 (4)
N9—C9	1.422 (3)	C635—C636	1.389 (3)
C2—H2	0.9500	C635—H635	0.9500
C4—C5	1.379 (4)	C636—H636	0.9500
C5—C6	1.401 (4)	C637—H63A	0.9800
C8—H8	0.9500	C637—H63B	0.9800
C9—C91	1.491 (4)	C637—H63C	0.9800
			
C6—S6—C61	100.88 (12)	O6—C62—C61	120.0 (2)
C634—O64—C637	118.2 (2)	C631—C62—C61	118.2 (2)
C6—N1—C2	117.4 (2)	C9—C91—H91A	109.5
C2—N3—C4	111.0 (2)	C9—C91—H91B	109.5
C8—N7—C5	103.8 (2)	H91A—C91—H91B	109.5
C8—N9—C4	105.1 (2)	C9—C91—H91C	109.5
C8—N9—C9	122.7 (2)	H91A—C91—H91C	109.5
C4—N9—C9	132.1 (2)	H91B—C91—H91C	109.5
N3—C2—N1	129.3 (3)	C636—C631—C632	118.2 (2)
N3—C2—H2	115.3	C636—C631—C62	123.1 (2)
N1—C2—H2	115.3	C632—C631—C62	118.6 (2)
N3—C4—C5	126.3 (2)	C633—C632—C631	121.3 (3)
N3—C4—N9	128.6 (2)	C633—C632—H632	119.4
C5—C4—N9	105.2 (2)	C631—C632—H632	119.4
C4—C5—N7	111.7 (2)	C632—C633—C634	119.3 (2)
C4—C5—C6	117.0 (2)	C632—C633—H633	120.3
N7—C5—C6	131.2 (2)	C634—C633—H633	120.3
N1—C6—C5	119.0 (2)	O64—C634—C635	124.0 (2)
N1—C6—S6	122.46 (19)	O64—C634—C633	115.0 (2)
C5—C6—S6	118.6 (2)	C635—C634—C633	121.0 (2)
N7—C8—N9	114.2 (2)	C634—C635—C636	118.9 (3)
N7—C8—H8	122.9	C634—C635—H635	120.5
N9—C8—H8	122.9	C636—C635—H635	120.5
O9—C9—N9	118.1 (3)	C635—C636—C631	121.2 (2)
O9—C9—C91	125.4 (2)	C635—C636—H636	119.4
N9—C9—C91	116.5 (2)	C631—C636—H636	119.4
C62—C61—S6	108.70 (19)	O64—C637—H63A	109.5
C62—C61—H61A	109.9	O64—C637—H63B	109.5
S6—C61—H61A	109.9	H63A—C637—H63B	109.5
C62—C61—H61B	109.9	O64—C637—H63C	109.5
S6—C61—H61B	109.9	H63A—C637—H63C	109.5
H61A—C61—H61B	108.3	H63B—C637—H63C	109.5
O6—C62—C631	121.7 (2)		
			
C4—N3—C2—N1	−1.2 (4)	C9—N9—C8—N7	176.4 (2)
C6—N1—C2—N3	1.4 (4)	C8—N9—C9—O9	7.5 (4)
C2—N3—C4—C5	0.7 (4)	C4—N9—C9—O9	−176.6 (3)
C2—N3—C4—N9	−179.0 (3)	C8—N9—C9—C91	−171.1 (3)
C8—N9—C4—N3	179.8 (3)	C4—N9—C9—C91	4.8 (4)
C9—N9—C4—N3	3.4 (5)	C6—S6—C61—C62	170.8 (2)
C8—N9—C4—C5	0.1 (3)	S6—C61—C62—O6	−7.7 (3)
C9—N9—C4—C5	−176.3 (3)	S6—C61—C62—C631	175.8 (2)
N3—C4—C5—N7	−179.5 (3)	O6—C62—C631—C636	160.4 (3)
N9—C4—C5—N7	0.2 (3)	C61—C62—C631—C636	−23.1 (4)
N3—C4—C5—C6	−0.4 (4)	O6—C62—C631—C632	−21.1 (4)
N9—C4—C5—C6	179.3 (2)	C61—C62—C631—C632	155.4 (3)
C8—N7—C5—C4	−0.5 (3)	C636—C631—C632—C633	0.7 (4)
C8—N7—C5—C6	−179.4 (3)	C62—C631—C632—C633	−177.9 (3)
C2—N1—C6—C5	−0.9 (4)	C631—C632—C633—C634	−1.1 (4)
C2—N1—C6—S6	178.9 (2)	C637—O64—C634—C635	9.6 (4)
C4—C5—C6—N1	0.5 (4)	C637—O64—C634—C633	−171.3 (2)
N7—C5—C6—N1	179.4 (3)	C632—C633—C634—O64	−179.2 (2)
C4—C5—C6—S6	−179.3 (2)	C632—C633—C634—C635	0.0 (4)
N7—C5—C6—S6	−0.4 (4)	O64—C634—C635—C636	−179.4 (2)
C61—S6—C6—N1	−8.5 (3)	C633—C634—C635—C636	1.5 (4)
C61—S6—C6—C5	171.3 (2)	C634—C635—C636—C631	−1.9 (4)
C5—N7—C8—N9	0.6 (3)	C632—C631—C636—C635	0.9 (4)
C4—N9—C8—N7	−0.5 (3)	C62—C631—C636—C635	179.4 (2)

### (2) 2-[(9-Acetyl-9H-purin-6-yl)sulfanyl]-1-(4-methoxyphenyl)ethan-1-one Hydrogen-bond geometry (Å, º)

**Table:**

*D*—H···*A*	*D*—H	H···*A*	*D*···*A*	*D*—H···*A*
C2—H2···O64^i^	0.95	2.48	3.375 (3)	157
C8—H8···O9^ii^	0.95	2.37	3.319 (3)	178
C61—H61*A*···O6^iii^	0.99	2.33	3.269 (3)	159

Symmetry codes: (i) −*x*+2, *y*+1/2, −*z*+1/2; (ii) −*x*−1, −*y*+1, −*z*+1; (iii) −*x*+1, *y*+1/2, −*z*+1/2.

### (3) 2-[(9-Acetyl-9H-purin-6-yl)sulfanyl]-1-(4-chlorophenyl)ethan-1-one Crystal data

**Table:**

C_15_H_11_ClN_4_O_2_S	*F*(000) = 712
*M**_r_* = 346.79	*D*_x_ = 1.603 Mg m^−^^3^
Monoclinic, *P*2_1_/*c*	Mo *K*α radiation, λ = 0.71075 Å
*a* = 5.9900 (4) Å	Cell parameters from 16488 reflections
*b* = 9.9169 (7) Å	θ = 2.5–27.5°
*c* = 24.3238 (17) Å	µ = 0.43 mm^−^^1^
β = 96.072 (2)°	*T* = 100 K
*V* = 1436.78 (17) Å^3^	Plate, yellow
*Z* = 4	0.13 × 0.06 × 0.01 mm

### (3) 2-[(9-Acetyl-9H-purin-6-yl)sulfanyl]-1-(4-chlorophenyl)ethan-1-one Data collection

**Table:**

Rigaku AFC12 (Right) diffractometer	3291 independent reflections
Radiation source: Rotating Anode	2677 reflections with *I* > 2σ(*I*)
Detector resolution: 28.5714 pixels mm^-1^	*R*_int_ = 0.050
profile data from ω–scans	θ_max_ = 27.5°, θ_min_ = 2.7°
Absorption correction: multi-scan *CrystalClear-SM Expert* (Rigaku, 20112)	*h* = −7→7
*T*_min_ = 0.809, *T*_max_ = 1.000	*k* = −12→12
18353 measured reflections	*l* = −31→31

### (3) 2-[(9-Acetyl-9H-purin-6-yl)sulfanyl]-1-(4-chlorophenyl)ethan-1-one Refinement

**Table:**

Refinement on *F*^2^	0 restraints
Least-squares matrix: full	Hydrogen site location: inferred from neighbouring sites
*R*[*F*^2^ > 2σ(*F*^2^)] = 0.035	H-atom parameters constrained
*wR*(*F*^2^) = 0.092	*w* = 1/[σ^2^(*F*_o_^2^) + (0.0466*P*)^2^ + 0.5802*P*] where *P* = (*F*_o_^2^ + 2*F*_c_^2^)/3
*S* = 1.02	(Δ/σ)_max_ = 0.001
3291 reflections	Δρ_max_ = 0.34 e Å^−^^3^
209 parameters	Δρ_min_ = −0.22 e Å^−^^3^

### (3) 2-[(9-Acetyl-9H-purin-6-yl)sulfanyl]-1-(4-chlorophenyl)ethan-1-one Special details

**Table:**

Geometry. All e.s.d.'s (except the e.s.d. in the dihedral angle between two l.s. planes) are estimated using the full covariance matrix. The cell e.s.d.'s are taken into account individually in the estimation of e.s.d.'s in distances, angles and torsion angles; correlations between e.s.d.'s in cell parameters are only used when they are defined by crystal symmetry. An approximate (isotropic) treatment of cell e.s.d.'s is used for estimating e.s.d.'s involving l.s. planes.

### (3) 2-[(9-Acetyl-9H-purin-6-yl)sulfanyl]-1-(4-chlorophenyl)ethan-1-one Fractional atomic coordinates and isotropic or equivalent isotropic displacement parameters (Å^2^)

**Table:**

	*x*	*y*	*z*	*U*_iso_*/*U*_eq_	
Cl64	1.32087 (7)	0.64277 (5)	0.10411 (2)	0.02661 (13)	
S6	0.38690 (7)	0.47381 (4)	0.33773 (2)	0.02313 (12)	
O6	0.6478 (2)	0.33342 (12)	0.26796 (5)	0.0267 (3)	
O9	−0.4321 (2)	0.65965 (14)	0.51215 (6)	0.0325 (3)	
N1	0.3921 (2)	0.73219 (15)	0.37483 (6)	0.0219 (3)	
N3	0.1372 (2)	0.82388 (15)	0.43621 (6)	0.0227 (3)	
N7	−0.0233 (2)	0.47859 (16)	0.41129 (6)	0.0242 (3)	
N9	−0.1394 (2)	0.65498 (15)	0.46015 (6)	0.0223 (3)	
C2	0.3079 (3)	0.82958 (18)	0.40503 (7)	0.0235 (4)	
H2	0.3800	0.9148	0.4041	0.028*	
C4	0.0447 (3)	0.70149 (19)	0.43464 (7)	0.0210 (3)	
C5	0.1118 (3)	0.59112 (18)	0.40522 (7)	0.0209 (4)	
C6	0.2939 (3)	0.61023 (18)	0.37446 (7)	0.0209 (4)	
C8	−0.1682 (3)	0.52134 (18)	0.44384 (7)	0.0233 (4)	
H8	−0.2836	0.4657	0.4554	0.028*	
C9	−0.2805 (3)	0.72219 (19)	0.49567 (7)	0.0247 (4)	
C61	0.5765 (3)	0.55799 (18)	0.29613 (7)	0.0231 (4)	
H61A	0.4932	0.6245	0.2714	0.028*	
H61B	0.6936	0.6065	0.3202	0.028*	
C62	0.6837 (3)	0.45309 (18)	0.26215 (7)	0.0215 (4)	
C91	−0.2263 (3)	0.8664 (2)	0.50911 (8)	0.0309 (4)	
H91A	−0.2262	0.9181	0.4748	0.046*	
H91B	−0.0778	0.8721	0.5302	0.046*	
H91C	−0.3393	0.9036	0.5312	0.046*	
C631	0.8399 (3)	0.50213 (18)	0.22261 (7)	0.0209 (4)	
C632	1.0125 (3)	0.41672 (18)	0.20934 (7)	0.0235 (4)	
H632	1.0258	0.3290	0.2250	0.028*	
C633	1.1644 (3)	0.45870 (19)	0.17350 (7)	0.0238 (4)	
H633	1.2840	0.4018	0.1653	0.029*	
C634	1.1368 (3)	0.58612 (18)	0.15003 (7)	0.0215 (4)	
C635	0.9650 (3)	0.67191 (18)	0.16161 (7)	0.0225 (4)	
H635	0.9483	0.7580	0.1445	0.027*	
C636	0.8181 (3)	0.63008 (18)	0.19851 (7)	0.0214 (4)	
H636	0.7018	0.6887	0.2075	0.026*	

### (3) 2-[(9-Acetyl-9H-purin-6-yl)sulfanyl]-1-(4-chlorophenyl)ethan-1-one Atomic displacement parameters (Å^2^)

**Table:**

	*U*^11^	*U*^22^	*U*^33^	*U*^12^	*U*^13^	*U*^23^
Cl64	0.0226 (2)	0.0260 (2)	0.0333 (2)	0.00033 (17)	0.01226 (17)	−0.00087 (18)
S6	0.0242 (2)	0.0180 (2)	0.0288 (2)	−0.00324 (17)	0.01066 (17)	−0.00245 (17)
O6	0.0289 (7)	0.0185 (7)	0.0338 (7)	−0.0011 (5)	0.0085 (5)	0.0000 (5)
O9	0.0269 (7)	0.0309 (8)	0.0423 (8)	−0.0021 (6)	0.0161 (6)	0.0031 (6)
N1	0.0208 (7)	0.0204 (8)	0.0250 (8)	−0.0025 (6)	0.0051 (6)	−0.0009 (6)
N3	0.0225 (7)	0.0200 (8)	0.0264 (8)	−0.0019 (6)	0.0061 (6)	−0.0013 (6)
N7	0.0215 (7)	0.0220 (8)	0.0300 (8)	−0.0045 (6)	0.0064 (6)	0.0005 (6)
N9	0.0189 (7)	0.0221 (8)	0.0269 (8)	−0.0017 (6)	0.0065 (6)	0.0017 (6)
C2	0.0222 (8)	0.0211 (9)	0.0279 (9)	−0.0047 (7)	0.0062 (7)	−0.0018 (7)
C4	0.0167 (8)	0.0253 (9)	0.0215 (8)	0.0010 (7)	0.0043 (6)	0.0017 (7)
C5	0.0190 (8)	0.0202 (9)	0.0240 (9)	−0.0014 (7)	0.0039 (7)	0.0006 (7)
C6	0.0183 (8)	0.0220 (9)	0.0227 (8)	0.0001 (7)	0.0042 (6)	0.0003 (7)
C8	0.0190 (8)	0.0238 (9)	0.0277 (9)	−0.0027 (7)	0.0046 (7)	0.0032 (7)
C9	0.0204 (8)	0.0272 (10)	0.0273 (9)	0.0006 (7)	0.0063 (7)	0.0018 (7)
C61	0.0255 (9)	0.0184 (9)	0.0270 (9)	−0.0021 (7)	0.0103 (7)	−0.0003 (7)
C62	0.0199 (8)	0.0215 (9)	0.0232 (8)	0.0017 (7)	0.0025 (6)	−0.0008 (7)
C91	0.0274 (9)	0.0307 (11)	0.0366 (11)	−0.0025 (8)	0.0134 (8)	−0.0072 (8)
C631	0.0193 (8)	0.0202 (9)	0.0235 (9)	−0.0001 (7)	0.0045 (7)	−0.0025 (7)
C632	0.0252 (9)	0.0182 (9)	0.0275 (9)	0.0031 (7)	0.0048 (7)	0.0009 (7)
C633	0.0202 (8)	0.0248 (10)	0.0270 (9)	0.0051 (7)	0.0050 (7)	−0.0022 (7)
C634	0.0187 (8)	0.0225 (9)	0.0239 (9)	−0.0017 (7)	0.0058 (7)	−0.0032 (7)
C635	0.0232 (8)	0.0173 (9)	0.0279 (9)	0.0008 (7)	0.0062 (7)	−0.0003 (7)
C636	0.0200 (8)	0.0181 (9)	0.0270 (9)	0.0018 (7)	0.0063 (7)	−0.0030 (7)

### (3) 2-[(9-Acetyl-9H-purin-6-yl)sulfanyl]-1-(4-chlorophenyl)ethan-1-one Geometric parameters (Å, º)

**Table:**

Cl64—C634	1.7433 (17)	C9—C91	1.495 (3)
S6—C6	1.7446 (18)	C61—C62	1.513 (2)
S6—C61	1.8038 (17)	C61—H61A	0.9900
O6—C62	1.217 (2)	C61—H61B	0.9900
O9—C9	1.203 (2)	C62—C631	1.493 (2)
N1—C2	1.344 (2)	C91—H91A	0.9800
N1—C6	1.344 (2)	C91—H91B	0.9800
N3—C4	1.333 (2)	C91—H91C	0.9800
N3—C2	1.337 (2)	C631—C636	1.398 (2)
N7—C8	1.306 (2)	C631—C632	1.400 (2)
N7—C5	1.395 (2)	C632—C633	1.389 (2)
N9—C8	1.389 (2)	C632—H632	0.9500
N9—C4	1.400 (2)	C633—C634	1.389 (3)
N9—C9	1.435 (2)	C633—H633	0.9500
C2—H2	0.9500	C634—C635	1.387 (2)
C4—C5	1.390 (2)	C635—C636	1.385 (2)
C5—C6	1.400 (2)	C635—H635	0.9500
C8—H8	0.9500	C636—H636	0.9500
			
C6—S6—C61	100.50 (8)	S6—C61—H61B	110.0
C2—N1—C6	117.46 (15)	H61A—C61—H61B	108.4
C4—N3—C2	111.27 (15)	O6—C62—C631	121.52 (16)
C8—N7—C5	103.57 (15)	O6—C62—C61	121.13 (16)
C8—N9—C4	105.53 (14)	C631—C62—C61	117.33 (15)
C8—N9—C9	123.49 (15)	C9—C91—H91A	109.5
C4—N9—C9	130.97 (15)	C9—C91—H91B	109.5
N3—C2—N1	129.31 (16)	H91A—C91—H91B	109.5
N3—C2—H2	115.3	C9—C91—H91C	109.5
N1—C2—H2	115.3	H91A—C91—H91C	109.5
N3—C4—C5	126.14 (15)	H91B—C91—H91C	109.5
N3—C4—N9	129.20 (16)	C636—C631—C632	119.40 (16)
C5—C4—N9	104.65 (15)	C636—C631—C62	121.92 (15)
C4—C5—N7	111.89 (15)	C632—C631—C62	118.68 (16)
C4—C5—C6	116.91 (16)	C633—C632—C631	120.80 (17)
N7—C5—C6	131.18 (16)	C633—C632—H632	119.6
N1—C6—C5	118.90 (16)	C631—C632—H632	119.6
N1—C6—S6	122.55 (12)	C632—C633—C634	118.25 (16)
C5—C6—S6	118.54 (13)	C632—C633—H633	120.9
N7—C8—N9	114.35 (15)	C634—C633—H633	120.9
N7—C8—H8	122.8	C635—C634—C633	122.19 (16)
N9—C8—H8	122.8	C635—C634—Cl64	117.76 (14)
O9—C9—N9	118.34 (17)	C633—C634—Cl64	120.05 (13)
O9—C9—C91	125.05 (17)	C636—C635—C634	118.94 (16)
N9—C9—C91	116.61 (15)	C636—C635—H635	120.5
C62—C61—S6	108.47 (12)	C634—C635—H635	120.5
C62—C61—H61A	110.0	C635—C636—C631	120.38 (16)
S6—C61—H61A	110.0	C635—C636—H636	119.8
C62—C61—H61B	110.0	C631—C636—H636	119.8
			
C4—N3—C2—N1	−0.7 (3)	C4—N9—C8—N7	0.2 (2)
C6—N1—C2—N3	0.7 (3)	C9—N9—C8—N7	179.77 (16)
C2—N3—C4—C5	0.2 (3)	C8—N9—C9—O9	−0.1 (3)
C2—N3—C4—N9	−178.24 (17)	C4—N9—C9—O9	179.26 (18)
C8—N9—C4—N3	178.49 (18)	C8—N9—C9—C91	−179.93 (17)
C9—N9—C4—N3	−1.0 (3)	C4—N9—C9—C91	−0.5 (3)
C8—N9—C4—C5	−0.24 (18)	C6—S6—C61—C62	177.77 (12)
C9—N9—C4—C5	−179.73 (17)	S6—C61—C62—O6	−4.5 (2)
N3—C4—C5—N7	−178.60 (16)	S6—C61—C62—C631	177.32 (12)
N9—C4—C5—N7	0.2 (2)	O6—C62—C631—C636	153.51 (18)
N3—C4—C5—C6	0.2 (3)	C61—C62—C631—C636	−28.3 (2)
N9—C4—C5—C6	178.95 (15)	O6—C62—C631—C632	−26.3 (3)
C8—N7—C5—C4	−0.1 (2)	C61—C62—C631—C632	151.92 (17)
C8—N7—C5—C6	−178.58 (19)	C636—C631—C632—C633	1.3 (3)
C2—N1—C6—C5	−0.1 (2)	C62—C631—C632—C633	−178.87 (16)
C2—N1—C6—S6	−179.32 (13)	C631—C632—C633—C634	−1.7 (3)
C4—C5—C6—N1	−0.2 (2)	C632—C633—C634—C635	0.5 (3)
N7—C5—C6—N1	178.25 (17)	C632—C633—C634—Cl64	−179.20 (13)
C4—C5—C6—S6	179.00 (13)	C633—C634—C635—C636	1.1 (3)
N7—C5—C6—S6	−2.5 (3)	Cl64—C634—C635—C636	−179.18 (13)
C61—S6—C6—N1	−11.73 (17)	C634—C635—C636—C631	−1.5 (3)
C61—S6—C6—C5	169.08 (14)	C632—C631—C636—C635	0.3 (3)
C5—N7—C8—N9	−0.1 (2)	C62—C631—C636—C635	−179.47 (16)

### (3) 2-[(9-Acetyl-9H-purin-6-yl)sulfanyl]-1-(4-chlorophenyl)ethan-1-one Hydrogen-bond geometry (Å, º)

**Table:**

*D*—H···*A*	*D*—H	H···*A*	*D*···*A*	*D*—H···*A*
C8—H8···O9^i^	0.95	2.31	3.262 (2)	176
C61—H61*A*···O6^ii^	0.99	2.40	3.354 (2)	162

Symmetry codes: (i) −*x*−1, −*y*+1, −*z*+1; (ii) −*x*+1, *y*+1/2, −*z*+1/2.

### (4) 2-[(9-Acetyl-9H-purin-6-yl)sulfanyl]-1-(4-bromophenyl)ethan-1-one Crystal data

**Table:**

C_15_H_11_BrN_4_O_2_S	*F*(000) = 784
*M**_r_* = 391.25	*D*_x_ = 1.758 Mg m^−^^3^
Monoclinic, *P*2_1_/*c*	Mo *K*α radiation, λ = 0.71075 Å
*a* = 6.0705 (4) Å	Cell parameters from 18109 reflections
*b* = 10.0668 (7) Å	θ = 2.5–27.5°
*c* = 24.3492 (17) Å	µ = 2.94 mm^−^^1^
β = 96.580 (2)°	*T* = 100 K
*V* = 1478.19 (18) Å^3^	Plate, colourless
*Z* = 4	0.15 × 0.10 × 0.02 mm

### (4) 2-[(9-Acetyl-9H-purin-6-yl)sulfanyl]-1-(4-bromophenyl)ethan-1-one Data collection

**Table:**

Rigaku AFC12 (Right) diffractometer	3346 independent reflections
Radiation source: Rotating Anode	2944 reflections with *I* > 2σ(*I*)
Detector resolution: 28.5714 pixels mm^-1^	*R*_int_ = 0.064
profile data from ω–scans	θ_max_ = 27.5°, θ_min_ = 2.6°
Absorption correction: multi-scan *CrystalClear-SM Expert* (Rigaku, 20112)	*h* = −7→7
*T*_min_ = 0.658, *T*_max_ = 1.000	*k* = −12→13
18171 measured reflections	*l* = −31→31

### (4) 2-[(9-Acetyl-9H-purin-6-yl)sulfanyl]-1-(4-bromophenyl)ethan-1-one Refinement

**Table:**

Refinement on *F*^2^	0 restraints
Least-squares matrix: full	Hydrogen site location: inferred from neighbouring sites
*R*[*F*^2^ > 2σ(*F*^2^)] = 0.056	H-atom parameters constrained
*wR*(*F*^2^) = 0.153	*w* = 1/[σ^2^(*F*_o_^2^) + (0.1178*P*)^2^ + 0.358*P*] where *P* = (*F*_o_^2^ + 2*F*_c_^2^)/3
*S* = 1.06	(Δ/σ)_max_ = 0.001
3346 reflections	Δρ_max_ = 2.91 e Å^−^^3^
209 parameters	Δρ_min_ = −0.92 e Å^−^^3^

### (4) 2-[(9-Acetyl-9H-purin-6-yl)sulfanyl]-1-(4-bromophenyl)ethan-1-one Special details

**Table:**

Geometry. All e.s.d.'s (except the e.s.d. in the dihedral angle between two l.s. planes) are estimated using the full covariance matrix. The cell e.s.d.'s are taken into account individually in the estimation of e.s.d.'s in distances, angles and torsion angles; correlations between e.s.d.'s in cell parameters are only used when they are defined by crystal symmetry. An approximate (isotropic) treatment of cell e.s.d.'s is used for estimating e.s.d.'s involving l.s. planes.

### (4) 2-[(9-Acetyl-9H-purin-6-yl)sulfanyl]-1-(4-bromophenyl)ethan-1-one Fractional atomic coordinates and isotropic or equivalent isotropic displacement parameters (Å^2^)

**Table:**

	*x*	*y*	*z*	*U*_iso_*/*U*_eq_	
Br64	1.32442 (5)	0.33869 (3)	0.10086 (2)	0.02971 (16)	
S6	0.38570 (14)	0.51673 (7)	0.33803 (3)	0.0278 (2)	
O6	0.6441 (5)	0.6537 (2)	0.26801 (11)	0.0318 (5)	
O9	−0.4299 (5)	0.3392 (2)	0.51161 (11)	0.0357 (6)	
N1	0.3870 (5)	0.2608 (3)	0.37506 (10)	0.0267 (5)	
N3	0.1320 (5)	0.1721 (2)	0.43630 (12)	0.0279 (6)	
N7	−0.0199 (5)	0.5157 (3)	0.41162 (11)	0.0274 (5)	
N9	−0.1388 (5)	0.3411 (2)	0.46032 (12)	0.0263 (6)	
C2	0.3003 (6)	0.1653 (3)	0.40499 (14)	0.0290 (7)	
H2	0.3683	0.0806	0.4038	0.035*	
C4	0.0420 (5)	0.2942 (3)	0.43479 (12)	0.0260 (6)	
C5	0.1121 (5)	0.4027 (3)	0.40513 (12)	0.0262 (6)	
C6	0.2904 (5)	0.3823 (3)	0.37469 (12)	0.0255 (6)	
C8	−0.1649 (5)	0.4742 (3)	0.44429 (12)	0.0274 (6)	
H8	−0.2771	0.5298	0.4559	0.033*	
C9	−0.2823 (5)	0.2762 (3)	0.49460 (13)	0.0279 (6)	
C61	0.5714 (5)	0.4314 (3)	0.29665 (13)	0.0278 (6)	
H61A	0.6861	0.3827	0.3209	0.033*	
H61B	0.4876	0.3666	0.2718	0.033*	
C62	0.6802 (5)	0.5351 (3)	0.26272 (12)	0.0267 (6)	
C91	−0.2352 (6)	0.1307 (4)	0.50629 (16)	0.0346 (7)	
H91A	−0.2341	0.0827	0.4713	0.052*	
H91B	−0.3506	0.0937	0.5268	0.052*	
H91C	−0.0904	0.1214	0.5283	0.052*	
C631	0.8358 (5)	0.4855 (3)	0.22393 (12)	0.0248 (6)	
C632	1.0055 (6)	0.5701 (3)	0.20986 (13)	0.0292 (6)	
H632	1.0178	0.6572	0.2250	0.035*	
C633	1.1556 (5)	0.5285 (3)	0.17420 (13)	0.0282 (6)	
H633	1.2725	0.5850	0.1657	0.034*	
C634	1.1296 (5)	0.4012 (3)	0.15118 (12)	0.0262 (6)	
C635	0.9596 (6)	0.3157 (3)	0.16372 (14)	0.0291 (6)	
H635	0.9435	0.2301	0.1473	0.035*	
C636	0.8144 (6)	0.3585 (3)	0.20067 (14)	0.0273 (6)	
H636	0.7005	0.3009	0.2100	0.033*	

### (4) 2-[(9-Acetyl-9H-purin-6-yl)sulfanyl]-1-(4-bromophenyl)ethan-1-one Atomic displacement parameters (Å^2^)

**Table:**

	*U*^11^	*U*^22^	*U*^33^	*U*^12^	*U*^13^	*U*^23^
Br64	0.0279 (2)	0.0250 (2)	0.0387 (2)	0.00019 (11)	0.01488 (15)	0.00074 (10)
S6	0.0299 (4)	0.0199 (4)	0.0361 (4)	0.0021 (3)	0.0142 (3)	0.0016 (3)
O6	0.0370 (13)	0.0205 (12)	0.0399 (12)	0.0015 (9)	0.0127 (10)	−0.0010 (8)
O9	0.0334 (14)	0.0301 (14)	0.0469 (14)	0.0016 (9)	0.0193 (11)	−0.0011 (9)
N1	0.0255 (13)	0.0225 (13)	0.0335 (12)	0.0028 (10)	0.0101 (10)	0.0010 (10)
N3	0.0316 (15)	0.0206 (13)	0.0328 (13)	0.0045 (10)	0.0095 (11)	0.0032 (9)
N7	0.0258 (13)	0.0214 (13)	0.0366 (13)	0.0036 (10)	0.0104 (10)	−0.0008 (10)
N9	0.0266 (14)	0.0206 (14)	0.0332 (13)	0.0012 (9)	0.0096 (11)	−0.0011 (9)
C2	0.0296 (16)	0.0222 (16)	0.0365 (16)	0.0043 (11)	0.0087 (13)	0.0015 (11)
C4	0.0260 (15)	0.0258 (16)	0.0277 (13)	−0.0002 (12)	0.0091 (11)	−0.0006 (12)
C5	0.0267 (16)	0.0202 (15)	0.0325 (14)	0.0035 (11)	0.0065 (11)	0.0008 (11)
C6	0.0249 (15)	0.0209 (14)	0.0316 (14)	0.0006 (11)	0.0070 (11)	0.0007 (12)
C8	0.0284 (16)	0.0214 (15)	0.0332 (14)	0.0025 (11)	0.0070 (11)	−0.0002 (11)
C9	0.0255 (15)	0.0255 (16)	0.0342 (14)	−0.0002 (12)	0.0097 (11)	−0.0009 (12)
C61	0.0298 (16)	0.0202 (15)	0.0358 (14)	0.0003 (11)	0.0143 (12)	−0.0001 (12)
C62	0.0269 (16)	0.0230 (15)	0.0312 (14)	−0.0026 (11)	0.0081 (11)	−0.0012 (11)
C91	0.0343 (18)	0.0275 (16)	0.0448 (18)	0.0033 (14)	0.0162 (14)	0.0072 (14)
C631	0.0251 (15)	0.0218 (15)	0.0287 (13)	0.0013 (11)	0.0079 (11)	−0.0003 (11)
C632	0.0317 (16)	0.0207 (15)	0.0364 (15)	−0.0020 (12)	0.0098 (12)	−0.0007 (12)
C633	0.0272 (16)	0.0246 (16)	0.0339 (14)	−0.0036 (12)	0.0086 (11)	0.0017 (12)
C634	0.0246 (15)	0.0232 (15)	0.0321 (14)	0.0001 (11)	0.0085 (11)	0.0015 (11)
C635	0.0318 (17)	0.0199 (14)	0.0376 (15)	−0.0025 (12)	0.0127 (13)	−0.0016 (12)
C636	0.0288 (16)	0.0187 (14)	0.0360 (15)	−0.0017 (11)	0.0098 (13)	0.0011 (11)

### (4) 2-[(9-Acetyl-9H-purin-6-yl)sulfanyl]-1-(4-bromophenyl)ethan-1-one Geometric parameters (Å, º)

**Table:**

Br64—C634	1.905 (3)	C9—C91	1.513 (5)
S6—C6	1.755 (3)	C61—C62	1.528 (4)
S6—C61	1.812 (3)	C61—H61A	0.9900
O6—C62	1.224 (4)	C61—H61B	0.9900
O9—C9	1.209 (4)	C62—C631	1.496 (4)
N1—C2	1.349 (4)	C91—H91A	0.9800
N1—C6	1.356 (4)	C91—H91B	0.9800
N3—C4	1.344 (4)	C91—H91C	0.9800
N3—C2	1.345 (5)	C631—C636	1.398 (4)
N7—C8	1.320 (4)	C631—C632	1.409 (5)
N7—C5	1.411 (4)	C632—C633	1.393 (5)
N9—C8	1.399 (4)	C632—H632	0.9500
N9—C4	1.404 (4)	C633—C634	1.400 (4)
N9—C9	1.431 (4)	C633—H633	0.9500
C2—H2	0.9500	C634—C635	1.404 (4)
C4—C5	1.402 (4)	C635—C636	1.398 (5)
C5—C6	1.395 (4)	C635—H635	0.9500
C8—H8	0.9500	C636—H636	0.9500
			
C6—S6—C61	100.33 (15)	S6—C61—H61B	110.1
C2—N1—C6	116.8 (3)	H61A—C61—H61B	108.4
C4—N3—C2	111.3 (3)	O6—C62—C631	121.7 (3)
C8—N7—C5	103.8 (3)	O6—C62—C61	121.1 (3)
C8—N9—C4	105.5 (3)	C631—C62—C61	117.2 (3)
C8—N9—C9	122.9 (3)	C9—C91—H91A	109.5
C4—N9—C9	131.6 (3)	C9—C91—H91B	109.5
N3—C2—N1	129.7 (3)	H91A—C91—H91B	109.5
N3—C2—H2	115.1	C9—C91—H91C	109.5
N1—C2—H2	115.1	H91A—C91—H91C	109.5
N3—C4—C5	125.4 (3)	H91B—C91—H91C	109.5
N3—C4—N9	129.2 (3)	C636—C631—C632	119.4 (3)
C5—C4—N9	105.4 (3)	C636—C631—C62	121.6 (3)
C6—C5—C4	117.3 (3)	C632—C631—C62	118.9 (3)
C6—C5—N7	131.5 (3)	C633—C632—C631	121.2 (3)
C4—C5—N7	111.1 (3)	C633—C632—H632	119.4
N1—C6—C5	119.4 (3)	C631—C632—H632	119.4
N1—C6—S6	122.1 (2)	C632—C633—C634	118.2 (3)
C5—C6—S6	118.5 (2)	C632—C633—H633	120.9
N7—C8—N9	114.2 (3)	C634—C633—H633	120.9
N7—C8—H8	122.9	C633—C634—C635	121.7 (3)
N9—C8—H8	122.9	C633—C634—Br64	120.7 (2)
O9—C9—N9	119.0 (3)	C635—C634—Br64	117.6 (2)
O9—C9—C91	125.1 (3)	C636—C635—C634	119.0 (3)
N9—C9—C91	115.9 (3)	C636—C635—H635	120.5
C62—C61—S6	108.2 (2)	C634—C635—H635	120.5
C62—C61—H61A	110.1	C635—C636—C631	120.4 (3)
S6—C61—H61A	110.1	C635—C636—H636	119.8
C62—C61—H61B	110.1	C631—C636—H636	119.8
			
C4—N3—C2—N1	1.4 (5)	C4—N9—C8—N7	−0.1 (4)
C6—N1—C2—N3	−1.6 (5)	C9—N9—C8—N7	−178.5 (3)
C2—N3—C4—C5	−0.5 (5)	C8—N9—C9—O9	−1.6 (5)
C2—N3—C4—N9	178.1 (3)	C4—N9—C9—O9	−179.6 (3)
C8—N9—C4—N3	−178.8 (3)	C8—N9—C9—C91	177.9 (3)
C9—N9—C4—N3	−0.6 (6)	C4—N9—C9—C91	0.0 (5)
C8—N9—C4—C5	0.0 (3)	C6—S6—C61—C62	−177.8 (2)
C9—N9—C4—C5	178.2 (3)	S6—C61—C62—O6	3.2 (4)
N3—C4—C5—C6	−0.1 (5)	S6—C61—C62—C631	−178.1 (2)
N9—C4—C5—C6	−179.0 (3)	O6—C62—C631—C636	−153.8 (3)
N3—C4—C5—N7	179.0 (3)	C61—C62—C631—C636	27.6 (4)
N9—C4—C5—N7	0.1 (3)	O6—C62—C631—C632	25.1 (4)
C8—N7—C5—C6	178.7 (3)	C61—C62—C631—C632	−153.6 (3)
C8—N7—C5—C4	−0.2 (3)	C636—C631—C632—C633	−1.5 (5)
C2—N1—C6—C5	0.8 (4)	C62—C631—C632—C633	179.6 (3)
C2—N1—C6—S6	179.6 (2)	C631—C632—C633—C634	1.9 (5)
C4—C5—C6—N1	−0.1 (4)	C632—C633—C634—C635	−0.8 (5)
N7—C5—C6—N1	−179.0 (3)	C632—C633—C634—Br64	178.7 (2)
C4—C5—C6—S6	−178.9 (2)	C633—C634—C635—C636	−0.8 (5)
N7—C5—C6—S6	2.2 (5)	Br64—C634—C635—C636	179.8 (2)
C61—S6—C6—N1	12.0 (3)	C634—C635—C636—C631	1.2 (5)
C61—S6—C6—C5	−169.3 (3)	C632—C631—C636—C635	−0.1 (5)
C5—N7—C8—N9	0.2 (4)	C62—C631—C636—C635	178.8 (3)

### (4) 2-[(9-Acetyl-9H-purin-6-yl)sulfanyl]-1-(4-bromophenyl)ethan-1-one Hydrogen-bond geometry (Å, º)

**Table:**

*D*—H···*A*	*D*—H	H···*A*	*D*···*A*	*D*—H···*A*
C8—H8···O9^i^	0.95	2.42	3.367 (4)	177
C61—H61*B*···O6^ii^	0.99	2.45	3.396 (4)	160

Symmetry codes: (i) −*x*−1, −*y*+1, −*z*+1; (ii) −*x*+1, *y*−1/2, −*z*+1/2.

### (5) 1-(3-Methoxyphenyl)-2-[(9H-purin-6-yl)sulfanyl]ethan-1-one Crystal data

**Table:**

C_14_H_12_N_4_O_2_S	*F*(000) = 624
*M**_r_* = 300.34	*D*_x_ = 1.487 Mg m^−^^3^
Monoclinic, *P*2_1_/*n*	Mo *K*α radiation, λ = 0.71075 Å
*a* = 7.6683 (5) Å	Cell parameters from 16870 reflections
*b* = 21.8004 (15) Å	θ = 2.5–27.5°
*c* = 8.4131 (5) Å	µ = 0.25 mm^−^^1^
β = 107.507 (2)°	*T* = 100 K
*V* = 1341.29 (15) Å^3^	Block, colourless
*Z* = 4	0.17 × 0.12 × 0.07 mm

### (5) 1-(3-Methoxyphenyl)-2-[(9H-purin-6-yl)sulfanyl]ethan-1-one Data collection

**Table:**

Rigaku AFC12 (Right) diffractometer	3063 independent reflections
Radiation source: Rotating Anode	2799 reflections with *I* > 2σ(*I*)
Detector resolution: 28.5714 pixels mm^-1^	*R*_int_ = 0.060
profile data from ω–scans	θ_max_ = 27.5°, θ_min_ = 2.7°
Absorption correction: multi-scan (*CrystalClear-SM Expert*; Rigaku, 2012)	*h* = −9→9
*T*_min_ = 0.724, *T*_max_ = 1.000	*k* = −28→28
17441 measured reflections	*l* = −10→10

### (5) 1-(3-Methoxyphenyl)-2-[(9H-purin-6-yl)sulfanyl]ethan-1-one Refinement

**Table:**

Refinement on *F*^2^	0 restraints
Least-squares matrix: full	Hydrogen site location: inferred from neighbouring sites
*R*[*F*^2^ > 2σ(*F*^2^)] = 0.033	H-atom parameters constrained
*wR*(*F*^2^) = 0.093	*w* = 1/[σ^2^(*F*_o_^2^) + (0.055*P*)^2^ + 0.4055*P*] where *P* = (*F*_o_^2^ + 2*F*_c_^2^)/3
*S* = 1.03	(Δ/σ)_max_ = 0.001
3063 reflections	Δρ_max_ = 0.30 e Å^−^^3^
191 parameters	Δρ_min_ = −0.37 e Å^−^^3^

### (5) 1-(3-Methoxyphenyl)-2-[(9H-purin-6-yl)sulfanyl]ethan-1-one Special details

**Table:**

Geometry. All e.s.d.'s (except the e.s.d. in the dihedral angle between two l.s. planes) are estimated using the full covariance matrix. The cell e.s.d.'s are taken into account individually in the estimation of e.s.d.'s in distances, angles and torsion angles; correlations between e.s.d.'s in cell parameters are only used when they are defined by crystal symmetry. An approximate (isotropic) treatment of cell e.s.d.'s is used for estimating e.s.d.'s involving l.s. planes.

### (5) 1-(3-Methoxyphenyl)-2-[(9H-purin-6-yl)sulfanyl]ethan-1-one Fractional atomic coordinates and isotropic or equivalent isotropic displacement parameters (Å^2^)

**Table:**

	*x*	*y*	*z*	*U*_iso_*/*U*_eq_	
S6	0.17185 (4)	0.41173 (2)	0.46144 (3)	0.02043 (11)	
O6	0.42208 (12)	0.47291 (4)	0.71480 (12)	0.0275 (2)	
O63	0.70355 (13)	0.67387 (4)	0.97072 (11)	0.0288 (2)	
N1	−0.06585 (14)	0.44463 (5)	0.16289 (13)	0.0227 (2)	
N3	−0.25987 (15)	0.37375 (5)	−0.03578 (13)	0.0240 (2)	
N7	0.01119 (14)	0.28258 (5)	0.30139 (13)	0.0211 (2)	
N9	−0.21099 (14)	0.26758 (5)	0.06007 (13)	0.0226 (2)	
H9	−0.2911	0.2481	−0.0214	0.027*	
C2	−0.19450 (17)	0.42912 (6)	0.01862 (15)	0.0247 (3)	
H6	−0.2451	0.4623	−0.0538	0.030*	
C4	−0.17933 (16)	0.32957 (6)	0.07248 (15)	0.0206 (2)	
C5	−0.04241 (16)	0.33841 (5)	0.22326 (14)	0.0195 (2)	
C6	0.01075 (15)	0.39879 (6)	0.26754 (14)	0.0194 (2)	
C8	−0.09352 (16)	0.24220 (6)	0.19916 (15)	0.0225 (3)	
H8	−0.0874	0.1993	0.2207	0.027*	
C61	0.21217 (16)	0.49315 (6)	0.44829 (15)	0.0213 (2)	
H61A	0.2602	0.5018	0.3537	0.026*	
H61B	0.0969	0.5163	0.4312	0.026*	
C62	0.35075 (16)	0.51187 (6)	0.61124 (15)	0.0207 (2)	
C631	0.39980 (16)	0.57789 (6)	0.63995 (15)	0.0205 (2)	
C632	0.52582 (16)	0.59345 (5)	0.79351 (15)	0.0208 (2)	
H632	0.5744	0.5626	0.8745	0.025*	
C633	0.57924 (17)	0.65409 (6)	0.82667 (16)	0.0230 (3)	
C634	0.50351 (18)	0.69940 (6)	0.70857 (17)	0.0259 (3)	
H634	0.5385	0.7410	0.7320	0.031*	
C635	0.37787 (18)	0.68404 (6)	0.55762 (17)	0.0265 (3)	
H635	0.3264	0.7152	0.4785	0.032*	
C636	0.32641 (16)	0.62298 (6)	0.52100 (16)	0.0238 (3)	
H636	0.2425	0.6123	0.4164	0.029*	
C637	0.7840 (2)	0.62823 (6)	1.09270 (17)	0.0301 (3)	
H63A	0.8724	0.6476	1.1886	0.045*	
H63B	0.8465	0.5976	1.0440	0.045*	
H63C	0.6882	0.6081	1.1291	0.045*	

### (5) 1-(3-Methoxyphenyl)-2-[(9H-purin-6-yl)sulfanyl]ethan-1-one Atomic displacement parameters (Å^2^)

**Table:**

	*U*^11^	*U*^22^	*U*^33^	*U*^12^	*U*^13^	*U*^23^
S6	0.02055 (17)	0.02018 (17)	0.01696 (16)	−0.00081 (10)	0.00019 (11)	−0.00014 (10)
O6	0.0294 (5)	0.0212 (5)	0.0250 (5)	−0.0002 (3)	−0.0021 (4)	0.0020 (3)
O63	0.0378 (5)	0.0234 (5)	0.0227 (5)	−0.0077 (4)	0.0053 (4)	−0.0043 (4)
N1	0.0231 (5)	0.0235 (5)	0.0196 (5)	0.0015 (4)	0.0036 (4)	0.0024 (4)
N3	0.0229 (5)	0.0282 (6)	0.0173 (5)	0.0029 (4)	0.0005 (4)	0.0022 (4)
N7	0.0203 (5)	0.0220 (5)	0.0178 (5)	0.0011 (4)	0.0009 (4)	0.0009 (4)
N9	0.0214 (5)	0.0242 (5)	0.0179 (5)	0.0001 (4)	−0.0005 (4)	−0.0024 (4)
C2	0.0250 (6)	0.0272 (7)	0.0185 (6)	0.0038 (5)	0.0016 (5)	0.0042 (5)
C4	0.0188 (5)	0.0249 (6)	0.0161 (5)	0.0011 (4)	0.0023 (4)	−0.0012 (5)
C5	0.0180 (5)	0.0233 (6)	0.0150 (5)	0.0015 (4)	0.0014 (4)	0.0006 (4)
C6	0.0179 (5)	0.0227 (6)	0.0166 (5)	0.0006 (4)	0.0038 (4)	0.0006 (4)
C8	0.0224 (6)	0.0222 (6)	0.0199 (6)	0.0004 (4)	0.0019 (4)	0.0000 (5)
C61	0.0206 (5)	0.0209 (6)	0.0203 (6)	−0.0011 (4)	0.0030 (5)	−0.0002 (4)
C62	0.0183 (5)	0.0225 (6)	0.0204 (6)	0.0000 (4)	0.0046 (4)	−0.0003 (4)
C631	0.0187 (5)	0.0219 (6)	0.0210 (6)	0.0000 (4)	0.0060 (4)	−0.0006 (4)
C632	0.0229 (6)	0.0202 (6)	0.0195 (6)	0.0003 (4)	0.0065 (5)	−0.0007 (4)
C633	0.0248 (6)	0.0240 (6)	0.0216 (6)	−0.0028 (5)	0.0091 (5)	−0.0030 (5)
C634	0.0298 (6)	0.0184 (6)	0.0312 (7)	−0.0018 (5)	0.0118 (5)	−0.0006 (5)
C635	0.0262 (6)	0.0228 (6)	0.0302 (7)	0.0021 (5)	0.0082 (5)	0.0053 (5)
C636	0.0208 (6)	0.0247 (7)	0.0241 (6)	0.0001 (4)	0.0042 (5)	0.0026 (5)
C637	0.0350 (7)	0.0315 (7)	0.0209 (6)	−0.0054 (5)	0.0039 (5)	−0.0024 (5)

### (5) 1-(3-Methoxyphenyl)-2-[(9H-purin-6-yl)sulfanyl]ethan-1-one Geometric parameters (Å, º)

**Table:**

S6—C6	1.7477 (12)	C61—C62	1.5162 (16)
S6—C61	1.8109 (13)	C61—H61A	0.9900
O6—C62	1.2219 (15)	C61—H61B	0.9900
O63—C633	1.3669 (15)	C62—C631	1.4887 (17)
O63—C637	1.4293 (17)	C631—C636	1.3949 (17)
N1—C6	1.3446 (16)	C631—C632	1.4025 (17)
N1—C2	1.3566 (16)	C632—C633	1.3871 (17)
N3—C2	1.3342 (17)	C632—H632	0.9500
N3—C4	1.3416 (16)	C633—C634	1.3975 (18)
N7—C8	1.3206 (16)	C634—C635	1.3846 (19)
N7—C5	1.3855 (15)	C634—H634	0.9500
N9—C8	1.3612 (15)	C635—C636	1.3963 (18)
N9—C4	1.3716 (16)	C635—H635	0.9500
N9—H9	0.8800	C636—H636	0.9500
C2—H6	0.9500	C637—H63A	0.9800
C4—C5	1.3954 (16)	C637—H63B	0.9800
C5—C6	1.3950 (17)	C637—H63C	0.9800
C8—H8	0.9500		
			
C6—S6—C61	100.77 (6)	H61A—C61—H61B	108.5
C633—O63—C637	116.88 (10)	O6—C62—C631	121.28 (11)
C6—N1—C2	117.22 (11)	O6—C62—C61	119.95 (11)
C2—N3—C4	111.58 (11)	C631—C62—C61	118.75 (10)
C8—N7—C5	103.95 (10)	C636—C631—C632	120.52 (11)
C8—N9—C4	106.19 (10)	C636—C631—C62	122.49 (11)
C8—N9—H9	126.9	C632—C631—C62	116.99 (11)
C4—N9—H9	126.9	C633—C632—C631	119.72 (12)
N3—C2—N1	129.01 (12)	C633—C632—H632	120.1
N3—C2—H6	115.5	C631—C632—H632	120.1
N1—C2—H6	115.5	O63—C633—C632	124.33 (12)
N3—C4—N9	128.37 (11)	O63—C633—C634	115.92 (11)
N3—C4—C5	125.80 (12)	C632—C633—C634	119.75 (12)
N9—C4—C5	105.83 (10)	C635—C634—C633	120.46 (12)
N7—C5—C6	132.92 (11)	C635—C634—H634	119.8
N7—C5—C4	110.17 (11)	C633—C634—H634	119.8
C6—C5—C4	116.90 (11)	C634—C635—C636	120.36 (12)
N1—C6—C5	119.44 (11)	C634—C635—H635	119.8
N1—C6—S6	122.55 (10)	C636—C635—H635	119.8
C5—C6—S6	117.99 (9)	C631—C636—C635	119.17 (12)
N7—C8—N9	113.85 (11)	C631—C636—H636	120.4
N7—C8—H8	123.1	C635—C636—H636	120.4
N9—C8—H8	123.1	O63—C637—H63A	109.5
C62—C61—S6	107.14 (8)	O63—C637—H63B	109.5
C62—C61—H61A	110.3	H63A—C637—H63B	109.5
S6—C61—H61A	110.3	O63—C637—H63C	109.5
C62—C61—H61B	110.3	H63A—C637—H63C	109.5
S6—C61—H61B	110.3	H63B—C637—H63C	109.5
			
C4—N3—C2—N1	−0.95 (19)	C4—N9—C8—N7	0.34 (14)
C6—N1—C2—N3	0.9 (2)	C6—S6—C61—C62	179.54 (8)
C2—N3—C4—N9	179.68 (12)	S6—C61—C62—O6	5.56 (14)
C2—N3—C4—C5	−0.74 (18)	S6—C61—C62—C631	−175.65 (9)
C8—N9—C4—N3	178.96 (12)	O6—C62—C631—C636	177.18 (11)
C8—N9—C4—C5	−0.68 (13)	C61—C62—C631—C636	−1.59 (17)
C8—N7—C5—C6	178.02 (13)	O6—C62—C631—C632	−2.42 (17)
C8—N7—C5—C4	−0.60 (13)	C61—C62—C631—C632	178.81 (10)
N3—C4—C5—N7	−178.84 (11)	C636—C631—C632—C633	−0.64 (18)
N9—C4—C5—N7	0.81 (13)	C62—C631—C632—C633	178.98 (11)
N3—C4—C5—C6	2.29 (18)	C637—O63—C633—C632	0.86 (17)
N9—C4—C5—C6	−178.05 (10)	C637—O63—C633—C634	−179.47 (11)
C2—N1—C6—C5	0.84 (17)	C631—C632—C633—O63	−178.68 (11)
C2—N1—C6—S6	−177.49 (9)	C631—C632—C633—C634	1.66 (18)
N7—C5—C6—N1	179.21 (12)	O63—C633—C634—C635	179.22 (11)
C4—C5—C6—N1	−2.24 (17)	C632—C633—C634—C635	−1.09 (19)
N7—C5—C6—S6	−2.39 (18)	C633—C634—C635—C636	−0.5 (2)
C4—C5—C6—S6	176.16 (8)	C632—C631—C636—C635	−0.96 (18)
C61—S6—C6—N1	−7.88 (11)	C62—C631—C636—C635	179.45 (11)
C61—S6—C6—C5	173.78 (9)	C634—C635—C636—C631	1.54 (19)
C5—N7—C8—N9	0.16 (14)		

### (5) 1-(3-Methoxyphenyl)-2-[(9H-purin-6-yl)sulfanyl]ethan-1-one Hydrogen-bond geometry (Å, º)

**Table:**

*D*—H···*A*	*D*—H	H···*A*	*D*···*A*	*D*—H···*A*
N9—H9···N7^i^	0.88	1.90	2.7715 (14)	171

Symmetry code: (i) *x*−1/2, −*y*+1/2, *z*−1/2.

## References

[bb1] Abdel-Aziz, H. A., Chia, T. S. & Fun, H.-K. (2012). *Acta Cryst.* E**68**, o2262. 10.1107/S1600536812028747 PMC3394045 22798910

[bb2] Agilent (2014). *CrysAlis PRO*. Agilent Technologies UK Ltd, Yarnton, England.

[bb3] Coles, S. J. & Gale, P. A. (2012). *Chem. Sci.* **3**, 683–689.

[bb4] Groom, C. R. & Allen, F. H. (2014). *Angew. Chem. Int. Ed.* **53**, 662–671. 10.1002/anie.201306438 24382699

[bb5] Heravi, M. R. P., Khouzani, L., Sadeghi, M. M. M., Zendehdel, M., Jackson, R. F. W. & Adams, H. (2009). *Anal. Sci. X-ray Struct. Anal. Online*, **25**, 43.

[bb6] Hübschle, C. B., Sheldrick, G. M. & Dittrich, B. (2011). *J. Appl. Cryst.* **44**, 1281–1284. 10.1107/S0021889811043202 PMC3246833 22477785

[bb7] Lambertucci, C., Antonini, I., Buccioni, M., Dal Ben, D., Kachare, D. D., Volpini, R., Klotz, K.-N. & Cristalli, G. (2009). *Bioorg. Med. Chem.* **17**, 2812–2822. 10.1016/j.bmc.2009.02.030 19282184

[bb8] Legraverend, M. (2008). *Tetrahedron*, **64**, 8585–8603.

[bb9] Legraverend, M. & Grierson, D. S. (2006). *Bioorg. Med. Chem.* **14**, 3987–4006. 10.1016/j.bmc.2005.12.060 16503144

[bb10] Liu, T. B., Jiang, W.-Q., Zou, J. P. & Zhang, Y. (2006). *Jiegou Huaxue (Chin. J. Struct. Chem.)*, **25**, 1019.

[bb11] Loghmani-Khouzani, H., Hajiheidari, D., Robinson, W. T., Abdul Rahman, N. & Kia, R. (2009*a*). *Acta Cryst.* E**65**, o2287. 10.1107/S1600536809033960 PMC2970099 21577679

[bb12] Loghmani-Khouzani, H., Hajiheidari, D., Robinson, W. T., Abdul Rahman, N. & Kia, R. (2009*b*). *Acta Cryst.* E**65**, o2441. 10.1107/S1600536809033121 PMC2970398 21577896

[bb13] Lynch, D. E. & McClenaghan, I. (2004). *Acta Cryst.* E**60**, o363–o364.

[bb14] Macrae, C. F., Edgington, P. R., McCabe, P., Pidcock, E., Shields, G. P., Taylor, R., Towler, M. & van de Streek, J. (2006). *J. Appl. Cryst.* **39**, 453–457.

[bb15] Masai, N., Hayashi, T., Kumazawa, Y., Nishikawa, J., Barta, I. & Kawakami, T. (2002). PCT Int. Appl. 2002081472.

[bb16] McArdle, P., Gilligan, K., Cunningham, D., Dark, R. & Mahon, M. (2004). *CrystEngComm*, **6**, 303–309.

[bb17] Rao, Z.-K., Zhang, S.-S., He, Y.-P., Zheng, Y.-T. & Li, C. (2007). *Acta Cryst.* E**63**, o3942.

[bb18] Rigaku (2012). *CrystalClear-SM Expert*. Rigaku Corporation, Tokyo, Japan.

[bb19] Sheldrick, G. M. (2015*a*). *Acta Cryst.* A**71**, 3–8.

[bb20] Sheldrick, G. M. (2015*b*). *Acta Cryst.* C**71**, 3–8.

[bb21] Spek, A. L. (2009). *Acta Cryst.* D**65**, 148–155. 10.1107/S090744490804362X PMC2631630 19171970

[bb22] Tunçbilek, M., Ateş-Alagöz, Z., Altanlar, N., Karayel, A. & Özbey, S. (2009). *Bioorg. Med. Chem.* **17**, 1693–1700. 10.1016/j.bmc.2008.12.050 19150600

[bb23] Yan, W.-L., Guo, Q., Li, C., Ji, X.-Y. & He, Y.-P. (2011). *Acta Cryst.* E**67**, o534. 10.1107/S1600536811003175 PMC3051611 21523182

